# A more equitable approach to economic evaluation: Directly developing conceptual capability wellbeing attributes for Tanzania and Malawi

**DOI:** 10.1016/j.socscimed.2024.117135

**Published:** 2024-07-14

**Authors:** Edith Chikumbu, Victor Katiti, Christopher Bunn, Elizabeth F. Msoka, Junious Sichali, Nateiya Mmeta Yongolo, Emma McIntosh, Blandina T. Mmbaga, Sally Wyke, Joanna Coast

**Affiliations:** a Malawi Epidemiology and Intervention Research Unit, P.O Box 46, Chilumba, Malawi; b Kilimanjaro Christian Medical University College, Kilimanjaro Clinical Research Institute, Box 2240, Moshi, Tanzania; c University of Glasgow, School of Social and Political Science, University Avenue, Glasgow, G12 8QQ, UK; d Kilimanjaro Christian Medical University College, Kilimanjaro Clinical Research Institute and Kilimanjaro Christian Medical Centre, P.O.BOX 2236, Moshi, Tanzania; e Kilimanjaro Clinical Research Institute, P. O Box 2236, Sokoine Road, Moshi, Tanzania; f Health Economics and Health Technology Assessment (HEHTA), School of Health and Wellbeing, University of Glasgow, Glasgow, G12 8TB, UK; g Health Economics and Health Policy @ Bristol, Bristol Medical School, University of Bristol, 1-5 Whiteladies Road, Bristol, BS8 1NU, UK

**Keywords:** Capability measure, Economic evaluation, Wellbeing, Qualitative methods, Tanzania, Malawi

## Abstract

Capability wellbeing can potentially provide a holistic outcome for health economic evaluation and the capability approach seems promising for African countries. As yet there is no work that has explored the evaluative space needed for health and care decision making at the whole population level and procedures that merely translate existing measures developed in the global north to contexts in the global south risk embedding structural inequalities. This work seeks to elicit the concepts within the capability wellbeing evaluative space for general adult populations in Tanzania and Malawi. Semi-structured interviews with 68 participants across Tanzania and Malawi were conducted between October 2021 and July 2022. Analysis used thematic coding frames and the writing of analytic accounts. Interview schedules were common across the two country settings, however data collection and analysis were conducted independently by two separate teams and only brought together once it was clear that the data from the two countries was sufficiently aligned for a single analysis. Eight common attributes of capability wellbeing were found across the two countries: financial security; basic needs; achievement and personal development; attachment, love and friendship; participation in community activities; faith and spirituality; health; making decisions without unwanted interference. These attributes can be used to generate outcome measures for use in economic evaluations comparing alternative health interventions. By centring the voices of Tanzanians and Malawians in the construction of attributes that describe a good life, the research can facilitate greater equity within economic evaluations across different global settings.

## 1 Introduction

Within all countries, decisions have to be made about how to allocate scarce health and care resources across the population. One approach to assisting these allocation decisions is the use of economic evaluation, in which the health and quality of life outcomes gained from interventions are related to the costs of that intervention, and then compared with the outputs per unit of cost from other interventions. Such an approach aims to enable resources to be used to obtain as much benefit as possible from each unit of resource. A dominant method for assessing health-related quality of life has formed in the global north, which utilises self-report questionnaires with preference-based valuations to estimate the impact of an intervention on health. Such instruments, include EQ-5D (Herdman et al., 2011) and SF-36 (Brazier et al., 2002); these have associated tariff values that are combined with length of life, to generate a Quality-Adjusted Life-Year (or QALY). Information about the cost per QALY gained through different interventions can be used in decision making by agencies such as the UK’s National Institute for Health and Care Excellence (National Institute for Health and Care Excellence, 2013).

While these preference-based measures have been used across sub-Saharan Africa, they are likely to contain implicit assumptions grounded in European and American approaches to valuing life (Pant et al., 2022). In addition, this dominant method focuses narrowly on health as the evaluative space (that is, the type of outcome of interest in evaluation) with health-related attributes (that is, the particular items or domains to be measured) and, as a consequence, may undervalue broader quality of life impacts. This has important equity considerations because different populations, genders and cultures may value attributes differently and the included attributes, levels and how they are valued will have implications for the subsequent ‘cost-effectiveness’ of interventions and the allocation of resources towards such interventions (Cookson et al., 2017).

An alternative economic approach is the capability approach. This derives from the work of Amartya Sen (1993) who aims to move away from narrow decision making foci (such as Gross Domestic Product (GDP) for example) as a basis for evaluation and towards an ‘evaluative space’ that can capture what a person is able to do and be in their life (Sen, 1993): the so-called ‘capability approach’. In the capability approach, the ‘doings’ and ‘beings’ that are important to capture within the evaluative space are focused on what a person has reason to value. The approach distinguishes between ‘functionings’ and ‘capabilities’, where functionings are what people actually do and are, and capabilities concern their freedoms or opportunities to achieve these functionings (Robeyns, 2017). The capability approach aligns with Sen’s emphasis on reducing inequalities (Sen, 1992), in part because it is designed to avoid the problem of adaptive preferences (preferences formed in light of what is available) that can come with the economist’s traditional approach that relies on utilities and preferences (Qizilbash, 2006).

Various theoretical contributions have explored the capability approach in health (Entwistle and Watt, 2013; Ruger, 2010; Venkatapurum, 2011). Measurement of capabilities for use in health economic evaluation has also been subject to growing interest (Anand and Dolan, 2005; Coast et al., 2008b; Cookson, 2005; Helter et al., 2019). In part this aligns with a desire to go beyond health in these evaluations, reflecting a desire for a wider conceptualisation of the benefits of health care (Mooney, 1998) and a greater concern with the social determinants of health within public health policy with a lens on ‘equity informed’ evaluations (Cookson et al., 2017). It also reflects growing confidence in the use of qualitative and participatory methods among health economists (Coast, 2017) such that they are able to design and conduct work to find out what people and patients think, and to build this into models for determining appropriate evaluative spaces. One of the earliest capability measures for use in economic evaluation came about precisely through this route, with capabilities being identified, through qualitative research that had been undertaken, as the relevant focus for a measure that could cross health and social care in the UK (Grewal et al., 2006).

Increased interest in capabilities among health economists has resulted in the development of measures for use in economic evaluation of health and care interventions. Some (Anand et al., 2005; Simon et al., 2013) derive from Nussbaum’s expert-driven set of 10 Central Human Capabilities which are assumed to be universal (Nussbaum, 2003), via work initially using items obtained from the British Household Panel Survey, but then supplemented and reduced (Anand et al., 2005; Lorgelly et al., 2015; Simon et al., 2013). Others have used research directly with relevant groups (primarily using qualitative methods) to identify the appropriate evaluative space (Greco et al., 2015; Grewal et al., 2006; Kinghorn et al., 2014). The ICECAP suite of measures has used these approaches to identify relevant evaluative spaces for assessing outcomes at different parts of the life course (Al-Janabi et al., 2012; Canaway et al., 2017; Grewal et al., 2006; Husbands et al., 2022; Sutton and Coast, 2014).

The applicability of the capability approach in the context of health care evaluation in African societies has been investigated through qualitative research on rural women’s health in Malawi (Greco et al., 2015), albeit amongst a specific demographic within the population. The approach was also used in the context of water, sanitation and hygiene in Mozambique (Ross et al., 2021) and among South Sudanese refugees in Uganda (van der Boor et al., 2024). Initial findings therefore suggest that the capability approach is promising for African countries in terms of providing an appropriate basis for economic evaluation, but as yet no work has explored the capabilities that are important for health and care decision making at the whole population level. One option, which is common in the economic evaluation area, is to translate existing measures, for example, health-related quality of life measures, but this is problematic where capabilities have been developed in one context and are assumed to be relevant in another: what people in one context value in terms of what they are able to do and be in their lives, may not be what is valued in a different context. Procedures that merely translate measures developed in the global north to contexts in the global south also risk embedding structural inequalities in approaches to evaluation through colonial imposition (Mignolo, 2012; Pant et al., 2022). Early exploration of the evaluative space in Tanzania incorporated the UK-developed ICECAP-A measure among arthritis patients (Kilonzo et al., 2023), but found that some attributes of the evaluative space, notably independence/autonomy and enjoyment, did not resonate with people in Tanzania.

This paper reports a qualitative study to elicit concepts for relevant evaluative space(s) for measuring capability wellbeing for general adult populations in Tanzania and Malawi for use in economic evaluation of health and care interventions. The study also aimed to systematically compare evaluative spaces across the two country settings, to determine whether they are sufficiently similar to enable the development of a single set of conceptual attributes for measuring capability wellbeing across both countries.

## 2 Methods

Semi-structured interviews were used within a subtle realist framework so that participants could articulate, in their own language, the aspects or attributes of their life that are important to them. To capture these conceptualisations, participants were asked to talk about what gave them good quality of life and what they valued in life. The terminology of “attributes” is used throughout, to retain consistency with previous work (Coast et al., 2008a).

### 2.1 Study setting

The research was carried out in rural and urban settings in Tanzania and Malawi. In Tanzania the rural area was Machame Ward, Hai District, a rural community on the slopes of Mount Kilimanjaro, and Moshi Municipality, an urban community on the flat lands surrounding the mountain. Both are located in Kilimanjaro Region, in the Northeast corner of Tanzania along the Kenyan border where agriculture provides about 75% of employment. In Malawi, the study was conducted in the Chilumba area in Karonga District, a rural community in the Northern Region of Malawi where livelihoods are based on smallholder farming and fishing and in Lilongwe, the capital of Malawi, an important economic and transportation hub.

### 2.2 Sampling and recruitment

In each setting, equal numbers of men and women aged over 18, including older and younger participants and those with and without chronic illness, were purposively sampled. This was because it was thought that a good life might mean different things to people in these groups. In Tanzania, sampling was from a population survey to investigate the burden of musculoskeletal conditions in the region (Kilonzo et al., 2023), and in Malawi, through searches of a database from a previous survey of the prevalence of chronic conditions in the Karonga district and Lilongwe (Price et al., 2018). All participants had given permission for further contact.

Potential participants were visited by enumerators in Tanzania and contacted by telephone in Malawi to check if they were interested in participation in this study and if so to meet with one of the research team (VK, EM in Tanzania; EC, JS in Malawi). Meetings took place at a local dispensary or at a street/village chairman office in Tanzania and at the potential participant’s home in Malawi. Researchers explained the study in participants’ vernacular language (Swahili in Tanzania and Chichewa or Chitumbuka in Malawi) using vernacular information sheets and encouraged participants to ask questions. A vernacular consent form was then read through with the participant and discussed. Written consent or thumb-print consent witnessed by a person of the participant’s choosing was given by all participants.

### 2.3 Data collection

Participants were interviewed by VK, EM, EC or JS in vernacular languages. After first gathering background information the topic guide (supplementary file 1) asked how participants spend their time in a single day. Within the framework of the day described, participants were asked what they enjoyed and what about these things they valued/or brought quality to their lives. They were asked about which aspects of life contribute to poor quality of life and what would improve their own quality of life. The interview then moved on to ask about specific aspects found to be important in other studies (Al-Janabi et al., 2012; Greco et al., 2015; Woodhouse and McCabe, 2018) including relationships with family and others, religion and spirituality, activities in employment or leisure, health and environment. Towards the end of the interview, participants were asked what they thought were the basic requirements for a good life.

Interviews were audio-recorded using digital recorders, stored and transferred using encrypted devices, transcribed verbatim by the researchers in Tanzania and by transcription assistants in Malawi whose first language matched that of each interviewee. They were then translated into English by those transcribing and anonymised for analysis.

### 2.4 Data analysis

Data analysis was conducted in two stages. First, independent analysis was conducted for each country following a common approach (Coast and Jackson, 2017). Researchers wrote brief summaries of the content of interviews conducted so that the research teams (VK, EM, NY, SW, JC in Tanzania; EC, JS, CB in Malawi) could gain initial familiarity with what might be important to participants. As transcripts became available, members of each research team read 5-10, noting down impressions and independently generating initial codes which focused on dimensions of what a good life was to participants. Following meetings in each country to discuss these codes, each team constructed a thematic coding frame, each theme of which could be seen as a potential attribute for a measure of capability wellbeing, and applied it to the full country dataset (VK,EM, SW in Tanzania using QDA Miner Lite and EC, CB in Malawi using NVivo). All members of the research team for each country then participated in writing analytic accounts for each theme incorporating quotes from participants and descriptive and interpretive narrative around those quotes. These were then combined into a detailed account for each country (supplementary file 2) (Coast and Jackson, 2017). Only once these independent country accounts were fully developed, were findings for the individual countries shared between the full authorship team across the two countries. To assess whether potential attributes of quality of life were similar or different in each country, the attributes derived in each setting were tabulated against one another, and the full team undertook detailed reading of the accounts before meeting to discuss and decide on the similarity of the content.

As the themes generated were so similar, a final thematic coding frame that considered small differences between countries was constructed, so that one full account could be provided here. VK, EC and SW applied the revised coding frame to the full, two country, data set, specifically examining potential differences by country, gender, age and chronic illness status. A final, full, analytic account was then written, noting where there were any differences between groups of participants (supplementary file 3). In this paper we report this final stage of analysis for both countries; supplementary files 2 and 3 provide the full separate and combined analytic accounts.

### 2.5 Reflexivity

The researchers who conducted interviews (VK,EM,EC,JS) are experienced social science researchers with masters qualifications who have conducted several other studies in these communities and who are based in research organisations familiar to community members. This enabled the building of rapport and trust, ensured culturally specific references and framings were communicated and understood and provided interviewees with a familiar point of contact to support accountability. It was noted that some, who were in particularly precarious circumstances with low income, could find it hard to talk about what made a good life. In those circumstances interviewers did not press, quietly waiting for a response and moving on as felt appropriate.

The analyst team occupy very different social positions to participants, having access to greater economic, cultural, and social capital and substantially different life experiences. These characteristics undoubtedly limited the extent to which the research team could fully understand the relevance of the attributes of a good life discussed by participants.

### 2.6 Ethical review

The study and its procedures were reviewed and approved by the Kilimanjaro Christian Medical University College Research Ethics and Review Committee (CRERC) in Moshi (KCMC/P. I/Vol.XI/2407). The National Institute for Medical Research Review Committee (NatHREC) in Tanzania (NIMR/HQ.R.8a/Vol.IX/3038), the National Health Sciences Research Committee in Malawi (ref: Protocol #21/08/2761:); Medical Veterinary and Life Sciences (MVLS) ethics committee at the University of Glasgow (UofG), UK (200180100).

## 3 Results

In total, 68 participants in Tanzania (34) and Malawi (34) were interviewed between October 2021 and July 2022. The interviews lasted between 14 and 84 min. Table 1 shows a breakdown of interviews undertaken in each setting and population group, and Table 2 summarises participant characteristics. Chronic conditions experienced by participants included HIV, diabetes, hypertension and arthritis/joint pain.

The independent analysis conducted for each country identified ten potential attributes in Malawi and eight in Tanzania. Table 3 shows the independently derived attributes and sub-themes for each country. The detailed reading of the content of these attributes (supplementary files 2 and 3) suggested that participants in Tanzania and Malawi had very similar views on what was important to them and thus that relevant evaluative spaces are similar in the two settings. The final, combined, list of attributes of capability wellbeing across both countries comprised: financial security; basic needs; achievement and personal development; attachment, love and friendship; participation in community activities; faith and spirituality; health; making decisions without unwanted interference. The sections below cover each capability wellbeing theme in turn, identifying when there were any differences between responses from participants in each country, living in rural or urban settings, or by gender, age or chronic illness status. If not mentioned, then no differences in response were identified. Data extracts are selected to illustrate how participants’ perspectives are linked to the emerging capability wellbeing themes. They are presented verbatim, with the use of ellipses to indicate removed text and are labelled first by country (Tz or Mw), then interview number, setting, gender and age.

### 3.1 Financial security

The importance of financial security ran through all participants’ accounts. Without prompting, participants spoke of the challenges of living with uncertainty about finances, both currently in the short term, and in relation to the future, in the longer-term. For many having good or poor quality of life depended on having money earned through employment or business:

I do shoe-shining and get money, children get clothing, food though to a small extent, but at least life continues … (Tz16, Urban, Male, 39)

Almost all participants spoke of the importance of having money to buy things and provide for life’s needs, and the difficulties that result from not having access to money in the short term. Both men and women spoke of the stress of not being able to properly care for children without money:

If I don’t have money, what can I feed my child? How can my child be assisted? So I feel very anxious. (Mw25, Urban, Female, 63)

I cannot be happy at all because I don’t have anything that I can depend on such as income or business, these children need exercise books, they need uniform … they need food, how will I get that? (Tz19, Rural, Male, 35)

Financial security was also valued because it enabled participants to balance between current spending and saving for the future, where savings could be used to invest in future wellbeing or to reduce uncertainty through mitigating future financial shocks.

I value to have good life, to have resources like livestock it’s what I wish to have … It’s keeping my things if I harvest, if I save they can help me later. (Tz34, Rural, Male, 53)

To ensure longer-term financial security, growing a business was seen as important, and those without financial security talked of wanting to have a business for income. Lack of capital, however, was seen as a major barrier to achieving financial security:

Availability of capital, when you have good capital, I think you will have a good future and get to the aim that you have (Tz22, Rural, Male, 36)

Participants with children spoke about the importance of investing in the future of their family and supporting children through education. Many also spoke about the importance of housing for longer-term financial security, with those who already lived in a family-owned home feeling that they had a measure of financial security and those in rented accommodation feeling that they would very much like to own a house:

I would like to have my personal house. As I said, I dwell in a rented house, so I wish or admire people who don’t pay rentals. (Mw22, Urban, Male, 30)

Sources of financial security were small business activity and employment. Education, through the ability to obtain more highly paid work, was also seen as a route to increased financial security. Children were seen as a source of current (for older people) or future (for younger people) financial security because they were expected to give money to care for their parents. A person’s health was also seen as an important enabler to allow people to work and thus to earn, and equally, poor health meant one couldn’t work and brought financial insecurity.

### 3.2 Basic needs

The basic needs theme focused on the immediate ability of participants to meet their basic needs, in contrast to the financial security theme, which focused on the challenges of living with uncertainty about access to finances. Basic needs were most often raised in direct prompting in interviews and considered to include food, housing, clothing, water and electricity. Finance was only relevant to this theme, in terms of its influence on participants’ ability to meet some of these essential needs. Being hungry is a familiar experience in both countries and food was often mentioned first:

The basic necessity for a good life is for a person to get at least three meals per day … in the morning, afternoon and at night. That way I see that at least it’s quality of life. One should get clothes and a good place to sleep that is quality of life … water I mean those basic services. (Tz6, Urban, Female, 33)

In both Tanzania and Malawi, it is common for people to grow food for themselves, and many discussed the value of knowing that even if they did not have money, at least they had food because they grew it themselves:

I live an enjoyable and comfortable life because I don’t lack food. I properly eat food because I have an opportunity of carrying out farming activities, on my own. I harvest and find everything that I lack in my life. (Mw28, Urban, Female, 51)

Clothing was seen as essential as a source of decency, and shelter was also commonly mentioned, as providing the basic security of having somewhere to live and sleep:

Having a house is one of the highest opportunities in my life. At my age, at the moment, I don’t get salary and pension … So, if it was not for putting up this structure, we should have been in the shit, but we are living properly. (Mw27, Urban, Male, 69)

Water was seen as essential, for showering and growing food. Some had to walk quite far to fetch water, and felt it made their life difficult. In Malawi in particular, electricity was seen as a basic need, because it made life much simpler and enabled other activities.

Electricity partly contributes to someone’s quality of life … we use it for cooking, ironing clothes, lighting, radios and watching TV, whenever we have an opportunity. However, we fail to do that due to unavailability of electricity. (Mw20, Urban, Male, 48)

### 3.3 Achievement and personal development

Participants spoke with pleasure about their achievements, both small and large.

They say that when they enter inside this house, everything looks tidy. The tidiness of my house, without basing on financial status, so they admire me. (Mw19, Urban, Female, 45)

For some people caring for one’s children well was a source of pride and achievement not only in providing for them but also in them being well-behaved, whilst older people, in particular, were able to look back and identify achievements in their lives.

I am glad that I have my house, a family eats well, children get to school to the good level to get employed also there is peace in the family they are things that have helped me in life and if God wishes all will be employed from my work. (Tz28, Rural, Male, 67)

Not being able to achieve their goals could make people feel bad, particularly those with children:

When I have planned something and fail to have it, I feel bad like why have I failed to reach my goal on time? If I don’t reach my goal my child will also fail and that way I keep on thinking on what to do best. (Tz22, Rural, Male, 36)

The term ‘development’ was often used, particularly in Malawi but in Tanzania too, to denote improvements to housing or infrastructure but also to oneself as an important aspect of achievement:

I wanted to develop myself in many things. At present, my life is better because since I started doing business and doing shares, I have done certain things, as of now. (Mw12, Rural, Female, 29)

### 3.4 Attachment, Love and friendship

Attachment was an important theme running through some participants’ accounts. Asked what they valued in life, participants focused on their close relationships.

My relationship with my wife, kids, my parents and my parents-in-law. They shape us together. (Mw11, Rural, Male, 34)

For some, the love of family was said to help overcome other difficult circumstances:

… it gives me a very good life even if I don’t eat, when there is love at home, I see my life is just fine. (Tz3, Urban, Female, 64)

Spouses were often valued for the enjoyment of bringing up children together, for emotional support, for the friendship they could offer and for making mutual decisions, although some married female participants also spoke of the difficulty if a husband drinks alcohol and uses a household’s income for himself. Women who were separated from partners were often bitter about a lack of support for their children. One Tanzanian participant spoke candidly about finding herself in conflict with her husband since she lost the intimacy of sexual relations after the menopause:

My husband and we listen to one another there is no any problem, we decide together, that is my happiness in my life (Tz23, Rural, Female, 44)

Children were highly valued, and those who still had living parents spoke of the support and love they offered, and wider families were discussed as a source of help and support, especially in Malawi. They could help if one were ill, offer financial help, and offer advice:

… when I hear that I am also called a mother, I have children, wow! I feel happy. (Tz6, Urban, Female, 33)

I tell my brothers whenever I lack something. I pay rentals on my own, but when I lack small things … my relatives assist me. (Mw29, Urban, Female, 33).

However, a few participants in both countries had difficult relationships with wider families, with causes of poor relationships seeming to relate to stigma associated with living with HIV and with being poor.

Participants mentioned friends or neighbours less often than family in both countries, usually in response to prompting. In Malawi, and to a lesser extent in Tanzania, participants mentioned chatting with or visiting friends as important to them. More generally, friends were seen as a less secure source of support, but could be a source of social capital, with relationships between friends being seen as mutually beneficial:

[Having friends] assists me in many ways because it’s like a social capital. For example, I have a cassava garden. When I harvest the cassava, I don’t sell it at the market. I use social capital. I just plan that I will sell my cassava in a certain area for two days and since I have friends in that area, it helps me to promote my business. People support me because of the way I chat with them. (Mw24, Urban, Female, 26)

### 3.5 Participation in community activities

Taking part in community activities was valued as part of what made a good life. Church communities were commonly said to provide support, with participating in church activities such as singing being valued:

Our church supports us reliably. We were members and church elders … We relate well with our fellow church members. (Mw32, Urban, Female, 68)

I like choir, going to the church so much, singing in the church (Tz31, Rural, Female, 57)

Community support was based on reciprocity. Contributing to community activities was seen as necessary in case one needed help oneself in the future, and activities like weddings and funerals were also seen as a way of supporting each other:

We cooperate on parties, if it’s a party we go and work together and contribute till we finish in the evening … in case of death when one has died you have to go and cooperate with them, to have strength, to see that they have people. (Tz33, Rural, Male, 65)

Supporting others was said to be affirming for those doing the helping, as this woman who visited sick people explained:

I feel that I have done a good job [when cheering the sick] because I have encouraged them and saved someone’s life. (Mw21, Urban, Female, 58)

The ability to take part in group activities was seen as valuable both for the person directly (for example through providing new ideas) and for the community (for example, through improving the local environment):

It’s good to undertake developmental activities because it helps the village. If there is a pothole on the road, we fill it, so that we can walk properly. (Mw15, Rural, Male, 26)

### 3.6 Faith and spirituality

Christianity is the main religion in both Malawi and Tanzania, although both countries also have sizable Muslim populations. Participants talked of a good life as synonymous with a life lived in faith and prayer, with the ability to worship bringing peace to participants, and what makes a good life being said to be a ‘godliness issue’:

It’s important to engage in religious activities in praying to God … when we earn a living that God set for us, that is what helps you pray to God. (Tz7, Urban, Female, 62)

They are godliness issues. I know that it’s God who makes everything possible, so it’s part of my spiritual life. Even if, I can face any kind of problems, but when I remember that God loves me, it gives me inner peace. (Mw18, Urban, Female, 30)

### 3.7 Health

A person’s health was seen as an important enabler of financial security, but good health was also valued *in its own right*, as a key component of a good life:

So, a good life is supposed to be healthy and enjoyable. The key thing is about having good health. (Mw19, Urban, Female, 45)

Some distinguished between good bodily health and the absence of stress:

My health is good … but there is stress because of how life is going, but physically it’s good. (Tz1, Urban, Female, 45)

The availability and cost of local health services was seen as an important source of good health. The lack of access to medicines that was perceived, particularly in Malawi, was noted as contributing to poor quality of life:

[I enjoy] health services being close to people together with government offices that are surrounding us, a problem can arise, and you get to them to explain. (Tz33, Rural, Male, 65)

So those things contribute to poor quality of people lives and I would love to see those things change. There should be availability of medication in hospitals. (Mw20, Urban, Male, 48)

The cost of health care, both in insurance and accessing treatment, worried participants, particularly in Tanzania:

If there are diseases like diabetic victims … you have to contribute [pay] and … many don’t afford to contribute so … people are dying because of lack of service that is what contributes to poor quality of life. (Tz5, Urban, Male, 49)

### 3.8 Making decisions without unwanted interference

Some participants, particularly in Malawi but also in Tanzania, said that being able to make decisions and live in their own way, was an important aspect of their lives:

May be what contributes to quality of life is making my own decisions not doing something for someone’s benefits. (Tz12, Urban, Male, 30)

It was not necessary to make decisions oneself; equally valued was making decisions with others. Decision making was usually a household matter, with consultation between members of the household before a decision is made, being valued:

I consult my husband at home. When we sat down, I told him that we are approaching rainy season, so what should we do? So, both of us gave our views, that due to shortage of money, we won’t manage to grow maize, so what should we do? We finally agreed that we should just grow groundnuts. (Mw21, Urban, Female, 58)

## 4 Discussion

Ideas of what made for a good life were very similar in Tanzania and Malawi, suggesting that the adult capability wellbeing evaluative space is similar in each. *Financial security* was a dominant theme in both settings amongst all participants and the ability to meet *Basic needs* was similarly important across participants, although elicited mainly through prompting. Being able to achieve one’s goals brought a valued sense of *Achievement and personal development*, and having close *Attachment, love and friendship* with family, especially children, could bring comfort and pleasure (with its absence bringing stress). *Participation in community activities, Faith and spirituality* (through love of God and praying), and *Being able to make decisions without unwanted interference*, either by oneself or with family, also appeared to be important. *Health* was valued as a source of financial security but also for itself, with participants expressing concerns about accessibility of health services and medicines critical to support health. That these two countries are both located in East Africa and have high levels of poverty (70% Malawi, 45% Tanzania), poor access to infrastructure such as electricity (14% Malawi, 43% Tanzania) and low levels of life expectancy (63 years Malawi, 67 years Tanzania) may account for some of the similarities in findings, particularly around the importance of *Financial security* and *Basic Needs* (World Bank, 2024).

Previous research developed a rural women’s capability measure for Malawi (Greco et al., 2015) and there are some clear similarities between the two pieces of work. Two of the six areas of wellbeing found by Greco et al. map directly to two of the attributes found here – Greco et al.’s ‘Economic security’ is closely related to *Financial security*, and their ‘Community relations’ is similarly close to the *Participation in Community Activities* found here. Three other attributes from Greco et al. relate to multiple of the attributes found here: ‘Physical strength’ to *Health* and *Basic Needs; ‘*Household wellbeing’ to *Making decisions without unwanted interference* and *Attachment, love and friendship;* and ‘Inner wellbeing’ most closely to *Faith and Spirituality* and *Achievement and personal development*, but also with ties to *Making decisions without unwanted interference*. One attribute identified through Greco et al.’s work, ‘Happiness’, did not appear as a separate attribute here, and is perhaps too close to the underlying notion of wellbeing itself to be perceived as just one component of wellbeing (Coast et al., 2012).

There are no comparable attempts to generate capability measures for Tanzania, but Woodhouse and McCabe have conducted work on wellbeing carried out with the Maasai of Northern Tanzania (Woodhouse and McCabe, 2018). They found wellbeing to be linked with concerns about future security, but in their work, this was more in relation to land than *Financial security*. Other similarities include autonomy in their work (relating to *Making decisions without unwanted interference* here), and a relational dimension that can be linked with both the *Attachment, love and friendship* and *Participation in community activities* attributes found here.

Relative to the ICECAP work conducted in higher income settings that uses similar methods to those pursued here, there were similarities and differences. Enjoyment, which appears in both ICECAP-A (Al-Janabi et al., 2012) and ICECAP-O (Grewal et al., 2006) did not feature as a separate attribute here, and the conceptual attribute of autonomy in ICECAP-A is expressed differently in Malawi and Tanzania. In the UK work, the focus was more individual, whereas in Malawi and Tanzania mutuality of decision making, often with others, was noted. Attributes not present in the UK work included *Faith and Spirituality*, and *Participation in Community Activities*. These are likely to relate to a greater commitment to the group, or collective endeavours, resulting from a more collectivist culture in the Malawian and Tanzanian settings (Hofstede, 2011), and to greater strength of religious feeling in most African countries (Pew Research Center, 2018). Most ICECAP measures have not explicitly included health-related attributes, with only the ICECAP-SCM (Supportive Care Measure) (Sutton and Coast, 2014) including attributes related to health, in terms of freedom from physical and emotional suffering. In the work in Tanzania and Malawi, however, health seemed to be of value for itself, and thus it was deemed appropriate to include it as a distinct attribute.

The sampling frames used here, which were databases constructed for surveys of long-term conditions in each country, brought both strengths and limitations. They enabled purposive sampling in rural and urban populations for gender, age and health state, so that the perspectives of a widely representative sample could be gathered. The samples are geographically limited, although they included rural areas with subsistence and small-business agricultural economies and urban areas where people are employed in private and public sector or run small businesses. Nevertheless, the typicality of findings to the wider Tanzanian and Malawian populations needs to be considered.

Researchers conducting interviews spoke the same language as participants, were familiar with the communities and were working in research organisations familiar to community members; we think this enabled them to build rapport and trust with participants. Although analysis was conducted in English, team members’ familiarity with the participants and ability to check back to the vernacular transcription, reduced the likelihood of meaning being lost in translation. The full research team included a wide range of perspectives including expertise in clinical and social science, with long experience of qualitative research and capability approaches, and in economic evaluation; nevertheless, they occupy very different social positions to participants and interpretations may be influenced by different assumptions based on prior experience.

It has been noted that evaluation theory, policies and practices, in common with many other areas of research, “derive from and have historically been dictated by Western frameworks” [Pant et al. (2022), p.3]. It is a strength of this research that it (i) draws on an underlying theory, the capability approach, developed as an alternative to standard Western frameworks through focusing on the freedom to pursue those things that a person has reason to value, and (ii) empirically, has aimed to centre the voices of Tanzanians and Malawians in the construction of attributes that describe a good life. The work thus provides an attempt to move away from the imposition of the values and norms encoded in measures constructed in the ‘global north’ on societies that do not share them, attempting to “default to the local gaze” [Ambibola (Abimbola, 2021), p.7]. Ultimately, it aims to contribute to greater equity in the economic evaluations that are conducted across different global settings.

This work highlights the areas of capability wellbeing that are important to populations in Tanzania and Malawi. It provides a starting point for evaluations conducted in a capabilities evaluative space by highlighting the impacts of health and care interventions and policy interventions around health and care, that such evaluations should capture. Currently, this work highlights the important conceptual attributes that should be factored into evaluation, but with further research and development including a valuation exercise it will provide a means of fully capturing outcomes in the capability evaluative space. This is important, where resources for health and care interventions are scarce, as it can ensure that these resources go towards interventions that produce the greatest improvement in capabilities within the population. There is also the potential to capture information about underlying capabilities and target interventions towards those in the population who have the greatest deficit in capabilities. It provides potential for evaluations of health and care interventions to go beyond health towards a broader conceptualisation of what it is important for people to be able to be and do in their lives. This began with Greco et al.’s work, but this work increases the potential significantly, by understanding impacts that are important across the whole adult population.

There are various avenues for further research arising from this study. To use these conceptual attributes in evaluation, some means is needed of collecting data that can indicate how people’s capabilities change in response to a health or care intervention, or in response to a policy change. In practice, this involves developing meaningful wording for the conceptual attributes, so that when individuals are asked questions about their capabilities, these underlying conceptual attributes are evoked (Coast et al., 2008a), and individuals are able to indicate how much of the capability they have. Although other ways of collecting data that do not reply on self-report are often advocated within the capability approach, it is difficult to conceive of means of collecting data about the extent to which individuals have these complex capabilities that do not rely on self-report. In determining methods for reporting capabilities, account will also need to be taken of both relatively low literacy levels in the different settings –around 82% in Tanzania and 68% in Malawi in 2022 (World Population Review, 2024) – and lack of familiarity with form filling (van der Boor et al., 2024). Interview administration may be required in some instances to mitigate these issues which is likely to add to costs of data collection in research studies.

As well as generating meaningful wording for reporting of capabilities, economic evaluation also requires some means of attributing relative importance to the achievement of the different capabilities: just because these eight capabilities were highlighted in the qualitative work, does not mean that they would all be considered equally important at a population level, so some means of assigning weights to reflect how important these capabilities are to Malawian and Tanzanian adults is also required. The ICECAP measures, developed and valued in the UK, have tended to use Best-Worst scaling approaches to generate these weights or scores, but this does not necessarily mean that a similar approach should be used for any Malawian/Tanzanian measures. Other approaches may better reflect the application of capability approaches in this setting, the cultural understandings in this group, and practical considerations around data collection. These issues need to be explored before any approach to generating weights is conducted.

Finally, it will be important to know that any eventual measures are acceptable to the population, that what is being measured is what researchers think that they are measuring, and that measures can adequately pick up the impacts of interventions that are experienced. This means that assessment of factors such as validity and responsiveness to change will be required.

This paper has described the development of conceptual attributes for use in evaluations of health and care interventions that are conducted within a capabilities evaluative space. An explicit focus was to address some of the structural inequalities in evaluation that arise when measures are imported to very different settings as a result of power and resource imbalances, with little consideration about their cultural appropriateness. This work found that the capabilities that were important to people in Tanzania and Malawi included health but went well beyond health. Any resultant measure using these capability well-being attributes would still, however, be suitable for evaluating health and care interventions because so many of the capabilities that emerged from this qualitative work can be influenced by poor health. For example: people may not seek health care because they are concerned about finances; physical health conditions may affect people’s ability to work & obtain financial security, participate in their communities and so on; mental and emotional health conditions may affect relationships, ability to make decisions without unwanted interference and so on. Use of a capability evaluative space in evaluation may provide a more rounded approach that can enhance efficiency, but through being grounded in Sen’s capability approach (Sen, 1992, 1993) takes equity as a more prominent objective.

## Supplementary Material

Appendix A. Supplementary data

Multimedia component 1

Multimedia component 2

Multimedia component 3

## Figures and Tables

**Table 1:** Participant interviews by sampling frame, across the two settings.

Sex	Men						Women						n
n	34						34						
Location	Rural			Urban			Rural			Urban			
n	17			17			16			18			
Age	18–35	36–55	56+	18–35	36–55	56+	18–35	36–55	56+	18–35	36–55	56+	
n	6	5	6	5	6	6	4	8	4	6	6	6	
Had chronic condition	2	4	6	1	2	2	1	6	3	1	1	4	68

**Table 2:** Summary of participants’ characteristics and mean duration of interviews.

Total number of interviews	68
Men	34
Women	34
Mean age men (yrs)	47.4
Mean age women (yrs)	46.4
Mean duration of interviews (mins)	35.0

**Table 3:** Comparison of potential attributes of capability wellbeing identified by independent analysis in Tanzania and Malawi, related to the final list of attributes for thematic coding.

Shared attributes for thematic coding		Malawi		Tanzania
Having Financial Security		To achieve financial stability		Financial Security
The experience of *having* financial security The challenges of living with uncertainty of financial resources available both currently and in the future				*Nature of financial security/insecurity*
		Education		Being able to HAVE financial security
		Employment		Short term
		Businesses		Longer term investment
		Land plots		Avoidance of financial implications of future shocks NOT being able to have financial security Short term Long term Anxiety about future shocks *Sources of financial security/insecurity* Business/farming prosperity Employment as a source of financial security Education as a source of financial security Infrastructure such as roads Children as a source of financial security
**Being able to meet basic needs**		**To access basic necessities**		**Basic Needs – the immediate meeting of needs**
Lists of basic needs and anything said about ability to meet OR NOT meet immediate basic needs		Electricity		*Nature of basic needs*
		Food		Being able to meet basic needs
		Water		NOT being able to meet basic needs
		Hygiene		*Sources of meeting basic needs*
		Phones		Having Water
		Vehicle - access to transport		Having Food Having food to eat
		**To feel secure and settled**		Being able to get enough food - from farming
		Shelter		Problems with climate change for farming
		Security		Business/Work Having Shelter Infrastructure - including education, health services, roads and electricity Having enough money to buy other things Other
**Achievement and Personal Development**		**To learn and develop as a person**		**Achievement and development**
Being able to achieve, or further develop, for oneself or one’s family		Education		*Nature of achievement - Being able to achieve*
		Human interaction		*Nature of achievement* - NOT being able to achieve
		Independence		*Sources of achievement* Farming Caring for children Fulfilling plans for Housing Family Working hard Educational infrastructure
**Attachment, love and friendship**		**To live harmoniously with family**		**Attachment - Love/friendship**
Ability to have and maintain attachments, including with close family (spouse/children), wider family, friends and neighbours		Negative influences		*Nature of attachment*
		Positive influences		HAVING attachment NOT having attachment *Sources of attachment (or lack of)* Having partner - wife/husband Having children Broader family Neighbours Friends Bereavement Illness and stigma from illness
**Being able to participate in community activities**		**To cultivate and maintain friendships in my community**		**Community Participation**
Taking part in community activities such as communal faith-based activities, weddings, funerals, sharing ideas with others and cooperating in activities with others in groups		Sharing advice		*Nature of Participation*
		Social capital		Being ABLE to participate
		Sharing of important information Negatives		Enjoyment Co-operation Self-respect/Social standing NOT being ABLE to participate
		**To enjoy my favorite pastimes** Sports		*Sources of community participation* Contributing to community activities
		Movies		Sharing ideas Being part of a group Taking part in activities Stigma from lack of children or poor health Alcohol use/drug use/bad behaviour
**Faith and Spirituality**		**To participate in my spiritual community**		**Faith and Spirituality**
Ability to express faith and pray though not the participation in communal religious activities such as going to church Include anything said about pleasure/peace etc about religious practice BUT not participation in religious community-based activities like choir singing or church going.		Sense of community		*Nature of spirituality*
		Express gratitude		HAVING spirituality Peace and emotional comfort
		Having roles in the congregation		Having God
		Moral compass		NOT having spirituality *Sources of spirituality* Involvement in religious activities Freedom to worship
**Health**		**To live a healthy life**		**Health Security**
Ability to lead a healthy life, with health valued in its own right rather than as a resource for financial security Including negative impact of poor health		Access to medical care		*Nature of health security*
		Being healthy		Being able to HAVE health security
		Implications of health		NOT being able to HAVE health security *Sources of health security* Absence of disease - In self Absence of disease - In family Looking after health Having access to health services & medicine
**Making decisions without unwanted interference**		**To make decisions without unwanted interference**		**Autonomy – making decisions without unwanted interference**
Ability to make decisions for their lives and that of their families, often in consultation with others Include anything about feeling good that can make decisions with on own or in consultation with others		Consulting other people		*Nature of autonomy*
		Individually		Being able to make own choices Nature of autonomy NOT being able to make own choices *Sources of autonomy* Own income Age

## Data Availability

Detailed accounts in supplementary data contain key data
