# Supplementary material for: A more equitable approach to economic evaluation: Directly developing conceptual capability wellbeing attributes for Tanzania and Malawi
Source: Soc Sci Med. Author manuscript; Available in PMC 2024 Nov 6. (PMC7616778; doi:10.1016/j.socscimed.2024.117135)
Supplement: Appendix A. Supplementary data [file EMS199746-supplement-Appendix_A__Supplementary_data.zip › 1-s2.0-S0277953624005884-mmc1.docx]

**Supplementary File 1. Capability Wellbeing in Tanzania and Malawi**

**Interview Topic Guide: Tanzania and Malawi**

## Aim of phase 1 interviews

- To explore people’s own perceptions of the dimensions of ‘a good life’ or quality of life
- To try to distinguish ways in which different factors are dimensions or components of ‘a good life’ or quality of life rather than influences / drivers / facilitators of that
- Within each dimension, to try to identify the elements that are unique to that dimension and those that are captured by other dimensions (to facilitate decisions about how components might be combined in the questionnaire)
- To begin to understand subjective levels in relation to each dimension

## Approach to interview

The interviews will be participant-led. The interview will open with broad questioning that invites the participant to generate and articulate in their own language the dimensions of a good life or quality of life that are important to them, and we will then follow these up. In other words, although the topic guide will ensure systematic coverage of the dimensions across interviews, the order and emphasis will be steered by the informant.

Therefore, interviews will open with a section that gathers some background information from which a pen-picture of the respondent can be produced. Following this would be a discussion of their current activities. This will be used to begin to develop a picture of the aspects of their lives which bring them pleasure or satisfaction, and will help to identify the dimensions of a good life or quality of life present and absent in their lives, which will guide the interview. The interview will be structured around exploring the dimensions raise by informants themselves, returning to the open questions to generate further dimensions, and finally introducing any factors that have not been raised.

**Identifying capability-based domains of quality of life in Tanzania and Malawi: a draft interview Topic Guide**

# Introduction

- Thanks for participation
- Explanation of the aims of the study
- Remind them interview is to be audiotaped
- Taking written/oral consent – reminder that they have signed/spoken consent form + give them a copy of consent form and participant information sheet to keep
- Explain that the interview can be stopped at any time
- Explain they can withdraw from the study at any time

**Background**

- Age
- Household composition (including marital status)
- Tenure/ how long lived there/ where they lived before
- Family/friends
- Carers/caring
- Employment
- Current health status

**Generating Positive Dimensions of QoL**

- How spend their time at moment
- What they enjoy about/gives them pleasure in their lives
  - in terms of what they do (e.g., looking after children, employment, social activities)
  - in terms of how they feel (e.g., feel satisfied at bringing up their children well)
- What it is about these factors that brings quality to their lives (e.g., what is it about looking after children that is important to them) –
  - how the factors contribute to their lives – what it is about them that is important
  - Exploration of how different factors contribute differently – e.g., comparing what friends versus family bring to QoL

**Generating Negative Dimensions of QoL**

- Aspects of life that contribute to poor QoL
  - things they would like to see change,
  - that they are not so happy with,
  - that they would like more or less of
  - reasoning behind factors that make QoL poor
- Comparison of own QoL with that of others, to ensure that all key QoL dimensions have been captured & understood.
- Key things that would improve their own QoL
- Key aspects of their lives that contribute to their own QoL

**Prompts for aspects not covered in the participant’s responses**

- Relationships with family – those living with, and those not living with
- Relationships with others (friends, neighbours, colleagues, carers)
- Religion/spirituality
- Activities including employment, hobbies, interests, contributions to their community, other activities
- Approaches to decision making: Independence/autonomy/collective/shared
- Health (physical & emotional/mental) & personal care (inc. health of others?)
- Surroundings (inc. housing & standard of living)
- Basic Needs
- Wealth/income
- Work (availability & conditions)
- Education, thought, reasoning

**Other issues**

- What opportunities/freedoms/choices do you value?
- What do you consider to be the basic necessities of a good life?
- Have the things that are important to you in your life changed?

# Ending

- Thanks for participation
- Ask if willing to take part in second interview (if appropriate)
- Remind the participant of the contact numbers found on the information sheet to use if they want to get in touch with researchers after the interview
