# Supplementary material for: A more equitable approach to economic evaluation: Directly developing conceptual capability wellbeing attributes for Tanzania and Malawi
Source: Soc Sci Med. Author manuscript; Available in PMC 2024 Nov 6. (PMC7616778; doi:10.1016/j.socscimed.2024.117135)
Supplement: Appendix A. Supplementary data [file EMS199746-supplement-Appendix_A__Supplementary_data.zip › 1-s2.0-S0277953624005884-mmc3.docx]

# Financial Security

Issues relating to financial security were a thread running through the accounts of almost all participants in both countries, both men and women and in all settings. Participants spoke the challenges of living with uncertainty about finances both currently in the short term, and in relation to the future, in the longer-term. For many having good or poor quality of life depended on having money:

I: Currently, what are the aspects that contributes to quality of your life? P: I think it’s income because if you don't have money it contributes to poor quality of life, so it’s struggling to work and get money. Tz 14 URBAN F 63

I: What do you think contributes to your quality of life? P: There is no meaningful job I: How does that contribute to poor quality of life? P: Because you fail to have income, it means you will live in problems. Tz 16 URBAN M 39

Money is earned through employment or business, which provides important things for oneself and one’s family:

I: Okay, what aspects of life that are currently contributing to your quality of life? P: What contributes is … the small job that I do I: How does a job you are doing contribute to quality of life? P: I do shoe-shining and get money, children get clothing, food though to a small extent but at least life continues I: What do you value so much in life? P: The work I am doing, I value it because it’s the one that makes a living for me now. Tz 16 URBAN M 39

Participants talked about experiences of living life with and without financial security, drawing on both their own experiences and those of others. The account of financial security first discusses the nature of financial (in)security as experienced by participants in the short-term, then the longer term, and then considers the sources of financial (in)security.

## Short-term financial (in)security

Almost all participants spoke of having money being important to buy things that are needed or wanted in a household:

A person needs to have a home, a good house with solid foundation, enough food, tap water and electricity should be available. Above all, she should earn money through employment or engage in a big capital business that can provide for things needed at the household. In so doing, things can work well for someone. Mw 25 Urban F 63

Money is used to buy the basic necessities for a family:

I: What impact does your business bring to your life? How does your business help you? P: After selling them, I buy food, soap and clothes for my children. Mw 14 Rural F

Not having money means one does not have those necessities:

There are many basic necessities that I want to have. However, for me to have those things, it requires many things. As I said, all the key things I need in my life are financially related. Even if, I can mention them, most of them are financially related. If I don’t have money, I can’t acquire most of those things. Mw 4 Rural M 67

Sources of income, such as employment or farming, are valued because of their ability to provide for such needs:

I: What else do you value? P: It's having a farm I: How? P: when you have a farm everything is accessed when you have yours you can plant anything like beans, maize, vegetables all can be obtained from that farm I: What else? P: Chicken and goats I: Why do you value them? P: Because if you have small problems, they can be solved instantly I: For example? P: If you sell you can buy an exercise book for a child, you give in a duck and you get Tz 26 RURAL M 55

I: What things do you have that you feel has greatly contributed to your quality of life? I don’t mean things that you desire, but things you already have which have greatly contributed. P: Things I do like business and employment in our family. They assist us in acquiring basic necessities for our lives. Mw 22 Urban M 30

And money, through employment, buys little luxuries that are important in life:

I: I want you to explain clearly. When someone is employed, how does she benefit from that employment? P: Okay, when someone is employed, she has opportunities of finding things that she needs during that time. For instance, she receives a salary after working. She can buy things like body lotion, soap. If she doesn’t have hair cosmetics, face powder like for us girls, she can manage to buy those things because she is working. Mw 30 Urban F 28

Equally, not having money, from employment or business means one lacks essentials which means one is unhappy, and lacks quality of life:

When I grew crops, I don’t quickly find customers to buy my farm produce, so I just keep them. It takes time for me to sell my farm produce. So, it becomes very difficult for me to find soap, salt and other things. Mw 7 Rural F 60

I: What else makes you unhappy now? P: What makes me unhappy now is that I don’t have money to get what I want such as eat well, dress nicely, getting a nice body oil. Tz 18 RURAL F 62

Lack of money, and higher prices, means one struggles to buy what is needed:

P: As I said, a person’s care and health hinges on diet, bathing and clothing. At present, things are difficult. Bathing soap, relish is expensive and cooking oil that we add to relish is also expensive. In other words, life has really changed, and we are in a difficult situation. Mw 20 Urban M 48

Women in particular spoke of not being able to properly care for children if one has no money:

I: You have cited lack of proper care for your children [as something that contributes to poor quality of life]. So, what actually cause this lack of proper care? P: Lack of proper care is as a result of difficulties in sourcing money. Mw 18 Urban F 30

P: I cannot be happy at all because I don’t have anything that I can depend on such as income or business, these children needs exercise books, they need uniform, they need shoes and more so they need food, how will I get that? there is nothing that makes me happy. All the time is hard to me, I keep on thinking ‘what will these children benefit with?’ Tz 19 RURAL M 35

Not being able to meet needs can bring the loss of self-esteem. For example, this older man does not like having to ask for help for essential needs:

The major thing that I am not so happy with, or can’t make me happy, is to beg from my friends. Tomorrow, I go to my neighbour and beg something from him. I think this is not a good thing. Normally, I don’t admire people who beg. [I don’t admire people] who walk along the road and knock at people’s doors, in order to beg for food and other things. I know that it happens to everyone, but it doesn’t give a good picture. In a village like this one, for me to be well known for causing problems because I don’t have anything that can assist me, is a very bad development. Mw 27 Urban M 69

And this older woman feels that she cannot visit her family because they treat her badly because of her poverty:

I: What makes you unhappy in your life, share with me P: I don’t get money to use, it’s very hard. Even if you decide to go and visit at your sister-in-law, the way they will be talking, you will just leave. Tz 18 RURAL F 62

Women in particular spoke of short-term financial security giving peace of mind that immediate needs could be met:

I: What else do you enjoy? P: The activities I am doing or? I: All that you enjoy P: May be I have planned my activity, I have gone to the market with success, that success gives me peace and happiness because get my needs, Tz 25 RURAL F 55

Just as not having money was feared and was stressful or meant they lack peace:

P: In my life, I don’t like to be broke. A person doesn’t need to go broke, so I hate to be broke because it makes me to have stress. For life to move on it’s because of money, so when I am broke it makes me to have stress. So, I don’t want this to happen to me. I don’t want to be broke. Mw 29 Urban F 33

I: Have you ever had stress in your life? Do you know stress? P: Yes. I: Have you ever had stress? P: Yes. I: What caused that? What makes you to reach a point of feeling anxious? P: I feel very anxious when I lack money or if I am not effectively running my business. I: So, what is the anxiety about? P: The anxiety comes in because as I am here, I have a child. So, if I don’t have money, what can I feed my child? How can my child be assisted? So, I feel very anxious. Mw 25 Urban F 63

As I said, there are many things that I am lacking. As I said, capital for starting a proper business that can help me to fulfil the things I lack. There are many things that a person needs in her life. I: Things like what? P: Things like food, school fees for children which I talked about. These are the things that are making me to really lack peace. Mw 25 Urban F 63

If there is lack of money in our family, we become worried because we think about what we would eat at our household or what we would do for the children. In so doing, a person can’t have quality life because she usually gets worried. It is that worrisome attitude that sometimes makes the disease to keep on multiplying because you don’t live a peaceful life. Mw 21 Urban F 58

The lack of money to have sufficient food for a family was also stressful for men some of whom spoke of wanting to provide adequately for their families:

I: In terms of your personal health and care for your family members, how does it affect your life? When you look at how you provide care to your family members? P: The way I care for my family members really affects me, because I don’t properly manage to provide enough things for my family due to lack of those things. I: Things like what? P: We have inadequate food sometimes, due to lack of money. If there is inadequate food at home, it really affects me when I look at the people whom I am caring. I ask myself that what I am I doing. When we lack certain things, I feel that my family is suffering a lot compared to my thoughts or contemplations. It really affects me when things are not okay. Mw 33 Urban M 68

On the other hand, having enough money for short term needs could bring pleasure

I: You have said that you enjoy spending time at the garden chatting with young men. So how does this give you pleasure? P: When I am at the garden, I sell the crops and earn a little something. I feel that it’s just the same with someone who goes early morning to work because I earn a little something. The women who are selling their tomatoes there have ordered them from me. In so doing, it gives me pleasure. Mw 17 Urban M 63

And business going well can make people feel good:

I: What do you enjoy in your life? P: I enjoy when things are going on well. I: How you know that things are going on well? P: When fisherman is able catching a lot of usipa and there is money kumtunda [markets where they sell usipa]. I: Can you explain, money kuntunda, from who? P: From people who buy our products. When Zambians come to buy things, they buy usipa from us and go with it. Sometimes people who go to South Africa also buy. I: You mean when you are successfully running your business, it makes you enjoy? P: I feel good. Mw 13 Rural M 41

Many people discussed the security of knowing that at least they had food to eat, often because they grew it themselves:

The other thing that I can say is that. In terms of opportunities that I have as an individual, I live an enjoyable and comfortable life because I don’t lack food. I properly eat food because I have an opportunity of carrying out farming activities, on my own. I harvest and find everything that I lack in my life. When I carry out farming activities, I live comfortably because I find everything, rather than buying everything because it’s a difficult and painful life. When I do those other things on my own, it becomes a good thing because I don’t feel anxious about what these people will eat or what will I give to visitors who have come unexpectedly. The little thing that I have or found is what I use to assist those visitors. Mw 28 Urban F 51

I: Is there anything [that helps in life]? P: I can say its struggling [farming] to get small needs like banana, fruits that helps me in life. Tz 34 RURAL M 53

But others felt they lack the food they wanted or needed:

When I lack money or food, life changes because I complain that I lack money and I don’t even have one tambala in my house. I also complain that I don’t have food in my house, so we will sleep on an empty stomach due to lack of nsima [flour] and I don’t know what to do in order to find nsima [flour]. Mw 5 Rural F 40

We also have inadequate food. There is an increase in commodity prices, so maize is expensive. It becomes very difficult for us to find food for feeding our 5 grandchildren. Mw 1 Rural M 80

Life for me is poor because you find you think maybe today if I had money I would buy even a quarter kilo of meat, you find you can't buy that meat, then you end up buying seafood/small fish so for me life is poor. Tz 3 URBAN F 64

Some spoke of lack of money leading to being able to eat only:

Income, you have no business to do [it’s okay but if] you have no income, no business to get income [it’s bad] because you eat once a day Tz 26 RURAL M 55

And others on the impact of eating only once a day on health and quality of life:

I: You talked about monetary issues. I want you to clearly explain how monetary issues affect your quality of life? P: When money is available in the family, we buy sugar and food that we eat during breakfast in the morning. The body becomes weak if we fail to eat in the morning as a result of lack of money. Later in the morning, I courageously kindle the fire [for preparing food] knowing that I bought okra, flour is available and then eat. In so doing, life becomes enjoyable. But if food is not available in the house, I can’t have quality life but poor quality of life. So, money is important in a person’s life. Mw 21 Urban F 58

## Long-term financial security

Financial security was also valued because it enabled participants to balance between current spending and saving for the future, where savings could be used to invest in future wellbeing or to mitigate future financial shocks.

A good job brings good income so that you can balance for home use and saving Tz 16 URBAN M 39

I: What do you value in your life? P: I value to have good life, to have resources like livestock it’s what I wish to have I: What else do you value? P: It’s keeping my things if I harvest, if I save, they can help me later. Tz 34 RURAL M 53

Growing a business was important, and those who did not have financial security talked of wanting to have a business for income.

When I observe the things that my friends have in their house. They have refrigerators, TV screens and good smartphones. I admire the things that my friends have, and I contemplate on how I can find the things that my friends have. I: You have talked about a refrigerator. How can your life change, if you can have a refrigerator? P: If I can have a refrigerator, I can use it for business. I: What kind of business? P: Business of selling soft drinks and locally brewed thobwa because they are on high demand. Mw 23 Urban F 40

I: Is there anything else you would like to see change apart from the disease? P: Yes, the other thing is about business. I would like my businesses to grow more than now. I should reach a certain level and be able to venture into other businesses. Mw 28 Urban F 51

Capital was seen as essential for good business, mentioned by men and women in all settings. Lack of capital was a major theme:

I: Do you have a business idea in your mind that you can do? P: Many of them. I: Can you mention one of them? P: I can be selling goods outside the country or sell them here. I can dry usipa there and then order other goods and deliver them, and then do other businesses. But, due to insufficient capital, I just do one side. If I can deliver the goods, I can earn profit and then do other business so that I shouldn’t lose capital. The other business can help me to earn money for household use, while the other business can help me to raise money for doing a certain thing. Like investing in a certain thing, like building a house and other thing. There are many things that I can do, but it’s due to insufficient capital. Mw 13 Rural M 41

I: Okay, what do you consider as basic necessities for good life? P: When you have a good for the family, you have a good job whether employed or you have got capital, they are good things that I value now. Tz 16 URBAN M 39

People felt life would be better with financial security which would be achieved with capital for business:

Availability of capital, when you have good capital, I think you will have a good future and get to the aim that you have Tz 22 RURAL M 36

I would like to see my life change. I need financial support or capital for engaging in a proper business. If I can obtain that capital, maybe my life can change. I can have quality life or an enjoyable life. Mw 25 Urban F 63

If I can have money, I can buy a plot, build houses for rent so that I can be receiving rentals. In so doing, I can be assisted in my everyday life (participant laughing). Yes, I also think of venturing into a huge capital business that involves travelling to other regions like ordering commodities at Mzuzu. I admire that if I can find a huge capital, I can go to order commodities in Mzuzu or Tanzania and sell them, I can have a good life. Mw 19 Urban F 45

The goods that can be bought with money was thought to enhance quality of life and had the potential to be used for business:

There are many things that are needed in a person’s life, and we can’t have everything. However, having a decent house, car, personal business and food at all times, can bring quality to my life. Mw 21 Urban F 58

Participants with children spoke particularly about the importance of financial security in investing in the future of their family,

In addition, my quality of life can improve, if I can have enough money because I can be able to properly assist my children as required. Mw 19 Urban F 45

I: What is good life to you? P: I mean, that life that I get whatever I want on time, food on time, health for my child and treatment on time and also if I don't have those I will setback my child and fail to reach his dreams. Tz 22 RURAL M 36

Supporting children through education was very important to parents and grandparents and borrowing for education was common:

I: What things would you like to see change in your life? P: I would like to see my life change. I need financial support or capital for engaging in a proper business. If I can obtain that capital, maybe my life can change. I can have quality life or an enjoyable life. At the moment, I don’t live an enjoyable life because I lack many things in my life. For instance, for me to raise money for fees in order for my girl to go to school now, I have just borrowed money from a young lady who stays in that house. I have borrowed from her so that we should just deduct it from her rentals. I have done this in order for my girl to go to school. Mw 25 Urban F 63

I use the loan to pay for my children school fees... Tz 21 RURAL F 34

SACCOS and get money that is how children go to school if I sell banana, avocado I add up from the SACCOS because I save there, if you save, they trust you and give you money Tz 27 RURAL F 50

And struggling to meet school or other education fees was a source of anxiety

In addition, I also pay fees for my child who is at the University, but I am still having difficulties in sourcing money up to now. He is a third-year student, but I am still having difficulties. Among the children I mentioned, my other child is at MUST University, my other child is at Chancellor College, my other child is at Secondary school and my other child is at Teachers Training College. All of them need financial assistance from me. I earn little money and with the increase in commodity prices, it’s challenging…. So, there are many things that contribute to poor quality of my life, I have mentioned few of them. Mw 20 Urban M 48

Many participants also spoke about the importance of longer-term financial security for investing in housing, with those who already lived in a family-owned home feeling that they had a measure of financial security. Investing in housing appeared largely to be related to reducing the need to pay rent, some also spoke about having tenants as a way of providing income, especially in Malawi urban. People who lived on their own plots were grateful they did not have to pay rent

This house properly assists me in my life because I don’t complain about certain things when I stay. I live comfortably and happily. We live comfortably in our house compared to a rented house. When we were living in a rented house, we were under pressure because we were contemplating that we would pay money for rent tomorrow. Our landlord was coming to knock at the door and ask us, don’t you know that the month has ended, so it wasn’t good for our lives. When we have our own house, we live comfortably and happily. Mw 28 Urban F 51

… already I don’t pay rent I am living in a family house so it don’t lose money on that part Tz 6 URBAN F 33

And those in rented accommodation would very much like to own a house:

Secondly, I admire to have my personal house. As I said, I dwell in a rented house, so I wish or admire people who don’t pay rentals. Mw 22 Urban M 30

the house we are living we also have our tenants; they gave us money… Tz 14 URBAN F 63

Finally planning, through savings to achieve financial security was important to avoid future shocks like unexpected health care costs, mentioned in particular in Tanzania:

Like if you lack capital you remain poor, because we expect you grow crops harvest sell some and remain with some, you keep livestock and sell some so when you lack and your health is not good you fail to take care of livestock, you fail to go to the market so you think of going to the hospital it's a lot of money if you don't have insurance, even if you have insurance at times you are told that we don't use insurance here so many challenges at times, the big thing is health. Tz 25 RURAL F 55

Others greatly valued the health insurance they had

I don't say that I have a very poor life, my life is on average because God gave me children and they are employed they know that there are parents, they have not forgotten us on what they get/earn they help us and the house we are living we also have our tenants they gave us money, our children have got health insurance for us we go to the hospital, I thank God

## Sources of financial security

### Business or farming prosperity

Having a business, particularly one that was doing well, was seen as a vital source of financial security for many, with no differences by gender, or rurality or age although there were more examples of the explicit link between a comfortable life and business success in Malawi than in Tanzania:

P: Things I have at the moment which support my life is the houses for rent. When I receive rentals during month end, I budget on things that I need to do and use it to order commodities in my grocery. ….. I am assisted by the money I get from rentals, and I am able to have food because of the rentals. Mw 25 Urban F 63

I: What are the key things that would improve your quality of life? P: If my businesses are doing well, it’s one of the things that can improve my quality of life. Mw 28 Urban F 51

I: For you to have quality life and live a comfortable life. P: It’s the business that I do. Mw 29 Urban F 33

I: What else do you enjoy? P: The activities I am doing or? I: All that you enjoy P: May be I have planned my activity, I have gone to the market with success, that success gives me peace and happiness because get my needs. Tz 25 RURAL F 55

if I get out in the morning and my business does well, I enjoy as I know that today I am sure of living well with my family Tz 2 URBAN M 45

I: Is it easy for you to earn K7000? P: It’s easy because I do business. When I sell beer and I have seen that its month end, I sometimes pay rent for 3 months. Mw 12 Rural F 29

I: You have said that you are a fisherman. Your father passed on, so how do you help your mother? P: When I come back from fishing and have earn money, I give her soap. It’s according to how God has given us. Tz 33 RURAL M 65

Having land where one could grow crops was very important for financial, and food security, especially mentioned by rural Tanzanian participants, although also by others. The food grown could be eaten or a surplus sold:

I: What else brings your quality of life? P: It’s farming that brings quality of life. Tz 34 RURAL M 53

I forgot to say that we also undertake farming activities. Starting from the past, we have been growing a lot of crops. So, when we harvest those crops and bring them here, my children get food here. // I: Do you harvest enough crops that you even sell them? P: Yes, we sell. I forgot to say this. We actually sell and earn money that we use for farming activities. People come to collect [buy] maize. Mw 32 Urban F 68

You have talked about growing crops. So, after you have harvested, do just use it for consumption or maybe you also sell? P: It depends on our preparation. We have prepared, monitored everything about the garden and harvested, so it depends on the crop yield. If I have harvested 50 bags, we can’t consume all of them (participant laughing). Although it attracts relatives. They say that ‘our relative grow crops so with this famine, maybe he can give us one bag?’. Anyway, if I harvest 50 bags, I can’t store all those bags for consumption. We sell some of them so that we should use the money to buy soap, sugar and other things. Mw 27 Urban M 69

P: The way I look after my cow I know I can solve all my problems, either you have your chicken lay eggs there I use, if I don’t have money for salt I will take my three eggs take them there at the shop and come back with salt, I don' t get a problem but saying that you keep on begging, no. Tz 23 RURAL F 44

P: I value things that I started with like vegetables and fruits that is what I started with, and they are the ones lifting me up. Tz 15 URBAN F 40

Yes, we harvest enough crops. Sometimes when we didn’t receive good rains, we face difficulties. But when we have cultivated crops, we have food. Mw 4 Rural M 67

For some people, multiple businesses were necessary to ensure their financial security

I earn money by engaging in small scale businesses and farming. I also have bicycle taxis that people use to carry customers. So, these are my sources of income. I: What type of farming do you carry out? P: I carry out different farming activities. Like last year, I grew maize only, but this year I will grow maize and soya beans. I also grow cassava. I: You talked about small scale businesses, so were you referring to selling of farm produce? P: Selling of farm produce and I also have bicycle taxis that young men use to carry customers. They give me a certain amount of money per week. Mw 24 Urban F 26

Although even two businesses, renting out properly and selling groceries from home was not enough for some:

I: What things do you have at the moment that greatly support your life? P: Things I have at the moment which support my life is the houses for rent. When I receive rentals during month end, I budget on things that I need to do and use it to order commodities. In my grocery. I don’t have many commodities [to sell] in my grocery, it’s here and there. The money I receive [from the grocery] is not enough ////. I am assisted by the money I get from rentals, and I am able to have food because of the rentals. Mw 25 Urban F 63

And others wanted to have a second line of business to increase their financial security:

I: Anything else? P: The issue of business for mills. Sometimes I can order second hand clothes and sell them at the market, it can also improve my life. Maybe, adding shops in order to sell groceries and other commodities that are needed to grow business. Mw 28 Urban F 51

Some spoke about the enjoyment or pleasure they got from their business or farming activities:

P: I enjoy most of the things I do. I also consider farming as an enjoyable thing to me, rather than just staying at home. For me to just stay at home now, I consider it as a useless thing. If I don’t have anything to do, it’s when I just stay idle. But for me to just stay idle when there is something that I need to do, I feel it’s unbeneficial. Most of the times, I like doing things, like farming because I enjoy. If one of my bicycle taxis is available, I personally go to carry passengers because I also enjoy it. I consider that as part of physical exercise and I also make money, so I enjoy. Mw 24 Urban F 26

I: What else makes you enjoy? P: When God blesses me to go to the farm to see how my plants are growing. Tz 26 RURAL M 55

I: Is there anything else that you value in your life? P: Its livestock, farming. I also like and value my village and country. Tz 20 RURAL M 77

I like preparing doughnuts in the afternoon. I invite some boys to mix the ingredients. After they have finished mixing, I roll them and invite a girl to fry the doughnuts. Mw 9 Rural F 58

Although one participant said she did not enjoy her business activities. Kachasu is home brewed alcohol:

I: Let’s talk about your business. You have said that you brew kachasu, so do you enjoy your business? P: I don’t really enjoy it because it’s a tiresome business. Regularly staying close to the fire is not good. But it depends on the money we earn because this business doesn’t require a big capital. It’s a kind of business that is run with little capital, but I don’t really enjoy it. I: So, you get exposed to the fire for a long-time? P: Yes. I regularly stay close to the fire. So, fire is harmful to our bodies. Mw 12 Rural F 29

Some participants said they would like to either start a business or grow their existing business if only they could get capital to invest:

P: I would like to have enough capital so that my business can grow. When the capital invested in a business is small, the business doesn’t grow. I: Do you mean that your business is not growing at the moment? P: Yes, because I invested a small capital in it. Mw 23 Urban F 40

P: As I said, there are many things that I am lacking. As I said, capital for starting a proper business that can help me to fulfil the things I lack. There are many things that a person needs in her life. I: Things like what? P: Things like food, school fees for children which I talked about. These are the things that are making me to really lack peace. Mw 25 Urban F 63

I: What do you wish to have to be like others? P: I wish to have a bigger capital to start a business that I can employ other people, mostly it’s capital to do something bigger than that. But the level I have is normal to support my family, children are not sent out of school [because I can’t pay] if a small problem comes, I can manage I: What are the things that would improve your quality of life? P: If I would get a bigger farm and own it and getting transport (car) to follow up things, to add livestock that I would use that car to bring food or have a bigger area to keep livestock that would help me more because I depend on livestock and farm I don't depend much in business, I don't believe so much because of climatic changes. Tz 28 RURAL M 67

P: The key thing is what I have already said. I should be doing business. I should find an opportunity of obtaining capital, so that I should be doing business while still working. I should run my business successfully and my family should go on well. Mw 26 Urban M 40

I: What else that you enjoy? P: Its only that I have no money, but I like to do business, if I get business to sell second hand clothes. Tz 34 RURAL M 53

I admire that if I can have a big starting capital for business, I can do business and reach a certain level. But since I don’t have those things, I have just accepted that I don’t have. Mw 2 Rural F 43

Some spoke about there being a difficult environment for business at the moment. For example, low commodity prices were difficult for small farmers:

I: What else can improve? P: Also, you have your cow and you milk, but selling price for milk is lower than the cost of taking care of it, it has to eat, there things the cow needs to eat to produce milk but looking at the money given by a milk buyer is small, these should be looked at. Tz 32 RURAL F 45

I: What crops do you grow? P: Maize. I: How is selling of your produce going about? P: Selling of our produce this year is very difficult due to reduced prices of maize. At the moment, I haven’t sold the maize due to reduced prices of maize. Mw 17 Urban M 63

I: So, there is nothing that you enjoy in your life now? P: Nothing because we do farming there is no rain, I do shoeshining no customers the situation is worse. Tz 16 URBAN M 39

Three rural participants thought their environment was good for business. For example, this rural Tanzanian farmer valued the land she was able to grow crops on, local roads to enable her to move her crops around, but also having a collection centre locally for her produce which meant she always had a market for her produce:

I: What do you value in life? P: Land when you have land where you live, you go for farming that is good. Another one also roads though not very good during rain - but a time like this it’s good, people move from one place to the other. Also … also chances we have there are markets if you want though I don't go everywhere, we also have centres to take milk for sale not waiting at home so when you keep livestock you are sure of selling milk, if its tomatoes you are sure of selling. Tz 25 RURAL F 55

And a rural Malawian participant also felt the area she lived in was generally good for business:

P: The thing that I enjoy about our area here is that money is found compared to other areas. You can have money, but you fail to do something with it. Sometimes you can do business, but it doesn’t run effectively. While in our area here, every business that someone can do runs effectively. Mw 12 Rural F 29

P: Living here is good because there many ways of generating income. If fishermen are not catching a lot of fish, those who brew kachasu [locally brewed alcohol] are busy with their business. So, if I can give them certain goods, I can be able to earn money. There are other areas that largely depend on fishing only, so if things are not working on that part, it becomes very difficult. We have many ways of generating income here. People who are working live around here. For instance, you [research centre] staff live here, hospital workers are here, teachers…. [ I: [You find piece work? P: Yes, piece work that people can do is found. There are many employees working in various sectors. Other areas are largely populated by farmers or fishermen. If strong winds can blow on the lake for two or three weeks, this can result in people lacking money. So, this area is good. Mw 13 Rural M 41

### Employment

Participants, both women and men and in both countries and in both rural and urban settings, saw employment as contributing to their financial security through the provision of wages.

I: What are the key things that you have at the moment which contribute to your quality of life? P: My husband’s employment and farming are our sources of income and assist in developing our household. Mw 28 Urban F 51

I: Is there anything else which you feel is needed in a person’s life? P: Things needed in a person’s life. I: Things that are needed for someone to have a good life. P: When someone is employed it’s also important. If someone is not employed, life becomes difficult. It requires someone to be employed. If someone is employed, things can work. Mw 32 Urban F 68

I: Can you tell me the things that you wish to be like others? P: To have work that can give me a good income I: For example? P: Like working in organisations, government or getting capital to be like them I: What do you think would improve your quality of life? P: Like I said having a good work that will provide a good income and life will automatically change. Tz 30 RURAL M 26

I: You said that you are 12 household members in total, so how do you make ends meet? P: We just rely on what we have earned that time. I: How do you earn it? P: My husband was working [still working], so we are assisted by the money he receives. He is about to retire. I: He is still working up to now? P: Yes. Mw 21 Urban F 58

Mainly, the work that I do helps me to earn something so that life should go on smoothly. Mw 11 Rural M 34

Even if work is not great, or pays too little, it is valued:

I: How about your work? P: I work but it's not that I am satisfied being there, I have plans I wish one day to be more than that though it helps to keep a family and my small things. Tz 30 RURAL M 26

May be because I am free to do the work I am doing and get something small, that is quality of life

because of the small job that I do… The work I am doing, I value it because it’s the one that makes a living for me now. Tz 16 URBAN M 39

Although two mentioned the difficult work environment they experienced,

I: How do you look at your working condition? How does it affect your life? P: I can say that it affects 50% of my life and lead to depression of my life because of what I get [how little the pay is] and how I relate with my superiors. This partly contribute to my life’s happiness because I have got friendships there, so they are like a family to me. Mw 26 Urban M 40

P: The salaries we get as security guards. /// Sometimes we work during public holidays, but they don’t pay us. Maybe, they also underpay us. When we complain about this, we don’t get any kind of assistance//// Security companies should have an established organization responsible for looking after the welfare of its workers so that if security guards face problems, they should be properly assisted, when they go. Mw 18 Urban F 30

Conversely, lack of employment was seen as contributing to financial vulnerability:

P: At the moment, I am not working. I just rely on piece works that people call me to fix electric faults. I: For you to stop working at those companies, what happened? P: Last time, I was working at ESCOM. It was a project for Meter Migration, so the project ended. I: Is it that you would want to go back and work for a company? What exactly is happening right now? P: I am still searching for a job. I want to get a permanent job. I just do these other things because I just stay idle, so I can’t refuse to do piece works. Mw 34 Urban M 30

I: Another thing? P: There is no employment life becomes hard. Tz 34 RURAL M 53

I: What are the aspects of your life that contribute to poor quality of your life? P: Its scarcity of jobs. I don’t secure jobs every day. I: What else? P: Nothing else. It’s only that. I: You have talked about employment. What are the other things that you would like to see change in your life? P: It’s only the issue of employment that I would like to see change. The other things are just okay. I: If you can get employed, how can your life change? P: I can’t say this in advance because it’s a thing that... I: As an individual, you contemplate on this. You don’t have that thing, but if you can have it, how can it change your life? P: It can change my personal life as well as for other people because I can be supporting my relatives or friends. Mw 34 Urban M 30

P: Like work, I could not get work even that of helping [people with small tasks] - but now I can get though [there is] not much [work]. Tz 13 URBAN M 19

I: Are there important things in life that have changed? P: Before shoeshining we were getting jobs to do even construction they were very many, we used to go and get back on the evening with money. Tz 16 URBAN M 39

### Education

Education was seen as a route to increased financial security, largely through the ability to obtain more highly paid work. Discussion of education in this way was often expressed through regrets about the person’s own lack of education and the potential it might have had to give them access to more highly paid employment.

I wish if I had the ability, I would have even gone for teaching so as I may become a teacher. Having education helps to a better thinking and you can get a well-paid job and you can thereafter sort your problems with your family Tz 21 RURAL F 34

what pains me more is education that I would have put in more efforts more than I think now to make sure I get to a certain point and be somebody, they are things that I think of and feel that I lost so much Tz 6 URBAN F 33

P: When I was a girl, the things that I wanted to happen in my life was about my education. I wanted to go further with my education and work hard in class so that I should be a nurse. //// Even in class, teachers could ask us that what kind of work I do [want] to do after completing my education. So, I was mentioning that I want to be a nurse, so they told me to work hard. I was also told about the subjects that I need to work hard on [in order to achieve that]. I was advised to be attentive, work hard, not to be playful and regularly miss classes. In addition, my parents, were also encouraging us to go to school and not to miss classes regularly. My mother really wanted us to go to school every time. She wasn’t allowing us to just stay at home, as it was happening with some of our friends. They could say that their mother had told them not to go to school in order for them to help her carry maize to the mill. My father and mother weren’t doing that. They were encouraging us a lot. However, we faced problems at the end when we started lacking or having difficulties in finding fees, so I just dropped out of school. I: In which class did you drop out? P: Standard 7. Mw 28 Urban F 51

P: Had it been that I completed my education, I wouldn’t have been thinking a lot about financial issues in my life. My children would have had quality life. I: Can you shed light on that? You have said that had it been that you completed your education, you wouldn’t have been thinking about financial issues. What do you mean? P: Had it been that I completed my education, my life would have changed. I: In what way? P: In such a way that I wouldn’t have been complaining about how I will look after my children. I would have gotten employed and become self-reliant. Mw 23 Urban F 40

I: What aspects of life that contributes to poor quality of life? P: I think its lack of education because if I had education I don't think I would have been living like this, it comes a time I need to go to school because I am good at cookery but you cannot ask for employment they will ask for the certificate that I don't have, if I had gone for hotel management it would have been easy or I want to go for tour guiding so you accept doing small activities. Tz 22 RURAL M 36

As we will see, children can be an important source of income for some, and educating children was seen as important by some participants because it would lead to them having a higher income and being able to help one out:

I: What else is needed in a person life? P: Having children who are educated is also a very important thing. If we educate children, we don’t lose. My children are educated and all of them are employed. They give us a little money to buy salt, cooking oil and other things because they are educated. Had it been that they were not educated, it would have been a problem. Mw 32 Urban F 68

I: How about education? P: Yes, I: How? P: when that child studies, by the grace of God he finishes school get employed, so already I will be saved in one way or another. Tz 32 RURAL F 45

One person wanted to be able to read for her own self-improvement – getting help from children or others at weekends:

I: What are the things you feel are important in your life at the moment? P: The other thing I feel is important is finding schoolbooks and reading them, so that I can be able to know other things. I should be writing. Some of my children or young men go to work [during weekdays], they find time during the weekend, so they can assist me. They can be giving me some reading tests and see how I am doing in terms of education. Mw 28 Urban F 51

Whilst another thought that more education for women was the key to family development and quality of life:

I: what aspects would improve quality of life? P: First we have to be with educative seminars educating women, when you educate a woman it's easy to improve life and a family and you teach how to take care of the family, quality of life starts from a family when you have educated a family that is the source of improving the economy and with that you can educate a neighbour and other people on what to do, before men women should be taught because women hold a family life and not men as we say. Tz 29 RURAL F 45

Two people mentioned education was important for quality of life without making the link with income explicit:

I: What aspects of life contributes to poor quality of life? P: First is laziness and alcoholism, you can get your money but if one in the family uses what you get different from what you agreed, also loosing hope because when you lose hope you fail to do your normal activities, education as well if you don't get good education it contributes to poor quality of life. Tz 25 RURAL F 55

### Children

Children clearly contribute to a number of different attributes of wellbeing, but it was clear that for some participants in both Malawi and Tanzania, and both men and women, one aspect of the value of children was as a source of current (for older people) or future (for younger people) financial security.

A number of older participants talked about how their adult children were able to provide them with resources if needed.

P: Key aspects of my life, it’s only my children. I don’t have anywhere else where I can depend on, it’s only my children. Two of my daughters were living in South Africa with their families. My other child works at the border. They clear vehicles before entering here in Malawi. My children are kind-hearted, and God should continue blessing them. They assist me in looking after my grandchildren. On my own, I can’t manage 32 Urban F 68

By the grace of God, I have three daughters who funds us. I can’t lie that I do any kind of business. The business is what my friend [wife] is doing here. Just to pass time, so that we should earn money for buying sugar and bathing soap. We have a grocery here. Otherwise, we don’t have any proper business that people can see and conclude that our friends are doing fine. We are just staying idle. It’s my children who mostly assist us. Mw 27 Urban M 69

I don’t pay anything. I need to be grateful for what my children are doing to me, as a parent. Mw 8 Rural M 73

What brings quality to my life is when I get money to eat and it is when the child comes back home with a little lucky, she tells me, mom I got this half kilogram of sugar take it that is when I see the quality of life, I get satisfied. Tz 3 URBAN F 64

Parents with both older and younger children also valued the contribution to financial security that the older children were able to make in providing for the younger ones.

P: I thank God that when time for school comes and you don't have anything [to buy younger children’s uniform and books] these older ones help you like school fees, uniform even in earnings they help so most of the time they help and are of help so much. Tz 25 RURAL F 55

One older child also saw the potential of this reciprocal relationship with his younger siblings.

My young brothers, I have to take [pay for them/support] them to school, if God blesses me, I will take them to school so that later they come to support me as well… My young ones going to school, I want them to go to school so that later they come and tell me, brother there is this place to stay come and stay here, you are now grown-up stop staying at people's places Tz 4 URBAN M 23

Parents with younger children saw their children as having the potential to help them in their older age.

I: But you have told me that you have a wife and two children? P: Yes, I: Don't you enjoy having them? P: I enjoy because i have children I: Why do you enjoy having children? P: I enjoy having children because everyone likes to have children so that they help me during my old age. Tz 16 URBAN M 39

I: What about the second thing? P: The second thing is what I explained about my children. If my children can be properly educated, they can have a brighter future and assist me in future, as well as other people. Mw 19 Urban F 45

Some parents were, however, disappointed that their children were not able to support them, or even led to greater financial vulnerability, rather than enhancing the parent’s financial security. A woman caring for her grandchildren wished her children would help:

I: Is there anything that you would like? P: In a family, children to change and have income, like to cater for my grandchild. The mother is in [city] [I would like to] make a call that there is this and that and they say, “mother don’t worry because I will be able to cater for that child.” Tz 23 RURAL F 44

expected my child to study and it reaches a time he/she rejects so when he/she comes back home he is dependent, what he does is not good at times he may become a thief or a use drugs, he doesn't bring anything home you keep on thinking what you get is used with no profit Tz 26 RURAL M 55

In one case, the child had died and so the parent was not able to access that source of financial security.

I had a child who died at times when I sit down remember and cry… I feel like if that child was alive, I wouldn't get problems like this, that child would have come to visit me, and you explain to him the problems and get support but now I have nothing Tz 18 RURAL F 62

### Health

Although good health was valued in its own right, a person’s own health was also seen as an important enabler to allow people to work and thus to earn. It was mentioned in both countries and by men and women, and by those with and without chronic illness, although the view was more commonly expressed amongst Tanzanian participants.

P: For someone to have a good life, she should rarely feel sick. It’s important when someone rarely feel sick because she does things with all her might. She can work and do other things. She can carry out household chores. On the other hand, when a person feels sick regularly, she can’t properly carry out household chores. Mw 32 Urban F 68

P: Even my health, i would thank God if I see changes I: What changes do you wish to see on your health? P: To be better, being health as before as health is a capital for working. Tz 14 URBAN F 63

I: Another thing that contributes? P: Also, diseases, because you find you are sick, and no one can go for earning then life must be poor. Tz 13 URBAN M 19

For some their health enabled them to work which in turn enabled them to care for their children.

P: Another thing is health, if I think of what made my father not to develop me [support his education] it was health and if [he had supported me] I would have not been failing everywhere. If I get sick now, I don't think my child will be happy. I have something like that in mind, I pray to God to give me strength to fight [work hard] as without health, without wellbeing I will not be able to struggle, I want when I have business, I open a bank account for him [his child] so that if I am not there one day he will use his bank account to continue. Tz 22 RURAL M 36

Good health was seen as enabling one to reach one’s ambitions or targets as well as earn or make money:

I: What are important things that you think would improve your quality of life? P: Things that would improve my quality of life I have said my good health, when I targets to do something, I should get success because my health is well and I have strength to do what I have targeted to do rather than being ill, I may not be able to fill full my set targets. Tz 25 RURAL F 55

And equally, poor health meant one couldn’t work and brought financial insecurity:

I: Do you think diabetes can contribute to poor quality of life? P: Yes, I: How? P: Work efficiency becomes low because sometimes you fail to do some activities like going for cow food you fail and [need to pay for] a labourer. Tz 32 RURAL F 45

I: Does this disease affect your daily routine activities, in anyway? P: Yes, when my blood pressure is high, I fail to carry out household chores. I feel body weakness and irregular heartbeat, so I don’t diligently do the work due to fatigue. In the past, when I was still having strength, I was ordering tomatoes and sell them, but I can’t manage this, at the moment. Mw 21 Urban F 58

I: What do you wish to change in the quality of life you have to have more quality of life? P: Like disease that I have, if I got treatment, I would change life. If I recovered from the diseases I have I would work more and change quality of life. Tz 20 RURAL M 77

I cannot work like before because now my body strength is getting down. Things which I use to do before such as woodwork, I cannot do it anymore because am sick

For some participants, there was also a concern with the costs of accessing healthcare and the implications for financial security.

going to the hospital it's a lot of money if you don't have insurance, even if you have insurance at times, you are told that we don't use insurance here so many challenges at times, the big thing is health Tz 25 RURAL F 55

One participant explained that paying for care for his wife’s illness meant he had to sell land and other capital, and now, because he is also sick, he cannot work:

I: What are the things that you think can improve your quality-of-life P: Business, I had wood business which my father left me with and selling meat business (slaughtering cows) I mixed that capital, and I had a huge capital. But since when my wife fell sick until when she died all the money was finished. I had a shop business I was able to do three business at once, my business was ruined by my wife’s sickness [cry] and I became poor. Now where I cannot work like before because now my body strength is getting down. Things which I use to do before such as woodwork, I cannot do it anymore because am sick. Tz 19 RURAL M 35

### Savings and Credit Cooperative Societies (SACCOS)

A few participants mentioned microfinance organisations like SACCOS as a source of financial security, through loans for investment.

P: The groups are the village banks that assist us. I: They assist you in what way? P: We borrow money from village banks. I: Have you joined those village banks? P: Yes. Mw 23 Urban F 40

I: What aspects would improve your quality of life? P: May be livestock because I am used to that because I did not go to school. [For example], adding cows [having more cows]. I was even telling my husband “If we could add a cow”, because one is not enough, on milk if one is pregnant we milk the other, that is what I think of, [I could go to] SACCOS and get money that is how children go to school. If I sell banana, avocado I add up from the SACCOS because I save there, if you save, they trust you and give you money. Tz 27 RURAL F 50

I: Are there things that other things that contribute to your quality of life? P: Involving my wife in groups when we get income from the farm, I involve her to go to groups I am also a SACCOS member there to invest there to be a able to get loan and to repay, life rotates it has a good rotation that does not affect so much for the family. Tz 28 RURAL M 67

### Infrastructure

Some Tanzanian participants from rural settings and a few Malawian participants from urban settings, talked about broader infrastructure as a factor influencing the ability to have financial security. This discussion was framed in terms of the ability to move goods to market, sell them and earn money, and the changes that were needed to infrastructure, particularly roads or utilities to enable this.

In a village is infrastructure when you come during rainy season those who have developed, they have their motorcycles moving people to the market, during the rain they cannot work and that is their employment… during rain a family will get problems Tz 24 RURAL M 18

P: Getting good roads, water these will contribute so much to improve our life, if you get safe water it helps to maintain our life rather than getting dirty water which will contribute to diseases and therefore our health will be in danger. If the climate is good, we will get rain and get good harvest. However, lack harvest and have no road to pass you will fail. Roads help to shorten a route - you don't take a long time [to get to market]. Tz 25 RURAL F 55

P: They should install electricity power at my residence. If they can install electricity power here, it means I can be able to earn money. For instance, I can have a maize mill that uses electric power. They installed a transformer long time ago, but they don’t connect electricity. If I can start using the maize mill, I can be able to earn money. Mw 17 Urban M 63

P: In terms of electricity at least there is stability, but the issue of water supply only. Maybe because different projects are underway to improve supply of water. We are having a problem of intermittent water supply, and this slightly affect people’s lives. Mw 24 Urban F 26

One felt that recent developments in infrastructure such as education and roads were good for their area:

P: In life the Nation is good, development is there compared to the past, graduates are there compared to the past I: For example? P: Roads were bad, car accidents but this time everywhere its tarmac roads, there are many things the government is doing, buying sheep we see that, modern rail ways there is development. Tz 28 RURAL M 67

### Working hard

When asked general questions about what makes a good or bad quality of life Tanzanian mainly older men and women from rural (though not urban) areas spoke about the importance of working hard implicitly meaning that working would bring financial security, or that not working hard would bring financial insecurity. This was not mentioned in Malawi.

I: What contributes to poor quality of life? P: Not working, if you don't work how will you have quality life? if you don't keep chickens [how] will you get money]…., if you don't keep cows? they are tiresome but you keep them to get life [income] there, how will you get if you don't keep [them]?, …if you don't work your life will drop. Tz 27 RURAL F 50

I: What are the things that have changed in life? P: I have not lived in poor life I am on average because I [work hard, if] you are lazy you can feel bad even living. Tz 28 RURAL M 67

I: What aspects of life that contributes to poor quality of life? P: It’s not working looking for something to help, if I don't struggle [strive] my life will be poor but if I work my life will be okay. Tz 33 RURAL M 65

I: Thank you, I like to know from you what are the things that contribute to poor quality of life? P: Life becomes poor when you don't work well or maybe you can have yields like milk to have good price or may be livestock to die from diseases or when you get diseases in your house they can also affect your life I: Are there other things that you think contributes to poor quality of life? P: Most it's that of not working but if a person works I don't think life will be hard. Tz 28 RURAL M 67

Although most people who talked about working hard as important for social security, some younger people did too, mentioning that younger people could take drugs and drink and not work to earn a living.

P: Most of the adolescents I interact with they are drunkard all the time, 24 hours they are on alcohol not doing anything, marijuana they are thieves because when you are not working you will engage in activities that are not official to earn a living so that is what I think contributes to poor quality of life to others. Tz 30 RURAL M 26

This view related to drink or drugs as a source of financial insecurity expressed by only a few Tanzanians almost entirely in relation to other people rather than themselves.

You find that you go for work and find others uses marijuana and when you get back home they tell you no money and while they were the one's taking drugs. If you ask that “we have been working together where has the money gone?” they tell you my money is finished I have no money now … we are three I have worked and other two have not worked, they wait for us to bring money for them as they keep on saying they have no money even when they work Tz 4 URBAN M 23

most of the time they are alcohol, you find on the evening has no food to cook of which if they had worked he/she would have got food Tz 31 RURAL F 57

The first I can say excessive drinking you find like in our street people from 30 years to 50 years engage so much in alcoholism that will contribute to poor quality of life, a person is working getting 15,000 is hard but getting 7,000 is possible, in running here and there he remains with 5,000, on the evening he goes to alcohol selling places and get drunk even if he remains with 3,000 he might not know where it is it can be stolen by others that contributes to poor quality of life Tz 24 RURAL M 18

### Inflation as a source of financial insecurity

Other sources of financial insecurity mentioned in both Tanzania and Malawi by both men and women was the problem of inflation, that is rising commodity and energy prices.

P: Every day, we find that there is an increase in commodity prices. So, the challenge is about sourcing money for buying those things. If I find those things and my family is happy because basic needs are available, it’s one of the things I can be happy with as a family man, because I am fulfilling my responsibility. Mw 26 Urban M 40

P: [Things] are very expensive, so we are failing to cope. Our salaries are not increasing, but prices of commodities keep on rising. Mw 18 Urban F 30

P: [The increase in utility bills] has affected us because prices are escalating, and the Kwacha is unstable here in Malawi. For instance, I still earn or source the same amount of money, but prices of commodities keep on rising every day. So, I am affected, in that way, because the earned money becomes insufficient when I want to spend it on our daily needs. Mw 22 Urban M 30

P: When you look at things are very expensive, getting money is also hard when you go to the shop and look at things its very expensive at times we fail to afford. Tz 31 RURAL F 57

P: Inflation that things keep on rising, and the items that they rise prices are basic things that we need, like soap is high, looking at cooking oil its very high, we used to buy salt at 350 now its 500, cooking oil a litre is 6000 something like that will you be happy?, why are they rising like that?, income is not enough there are school children, they are getting sick, weeping. Tz 23 RURAL F 44

It is a lot of things that make life miserable because if the prices of things have gone up, we used to buy a spoon of cooking oil at five hundred, now a spoon is one thousand shillings, now when you sell corn you make profit of one thousand five hundred shillings, the one thousand you buy cooking oil alone, you still haven't bought vegetables/sauce for home, in fact my life is getting poor. Tz 3 URBAN F 64

I: Mention for me another reason that contributes to poor quality of life #00:00:01-2# P: Another reason is Inflation #00:00:01-2# I: How? #00:00:01-2# P: For example we used to buy one litre of cooking oil for three thousand, now a litre is eight thousand when you look at baking powder we used to buy a kilo at one thousand five hundred, now a kilo is two thousand two hundred that contributes to life being hard #00:00:01-2#. Tz 26 RURAL M 55

### Climate change as a source of financial insecurity

In Tanzania, rural and some urban participants talked of climate change as a source of financial insecurity. The rural economy in this part of northern Tanzania is based on agriculture and low rainfall was severely impacting on crop yields.

P: Climate also as you see maize are dry that we were depending on that, it has been there is no rain, we waste a lot of time in the farm but there is no success. Tz 31 RURAL F 57

P: This farming like renting a farm, you buy seeds, fertilizer so that to get harvests but there is no rain, like now I have rented three acres at 750,000, you have bought seeds for three acres, you have bought fertilizer its high, you have planted seeds down there but they don't grow that is a challenge, you find that you have borrowed that money from the SACCOS you are supposed to repay that is a challenge already. Tz 32 RURAL F 45

I: I would like to know, what do you think contributes to poor quality of life? P: I think there are things that contributes, my personal life challenges on inflation on cooking oil, rain getting late I have used much costs to prepare a farm and maize are drying up. Things like that contributes to my poor quality of life. Tz 30 RURAL M 26

I: Is there anything that contributes to poor quality of life? P: I think life is contributed by climate as well, if there would be rain at least life would have been better because we would do farming, harvesting and living well. Tz 16 URBAN M 39

Climate change was not mentioned by Malawian participants as a source of insecurity. Although even in urban areas every spare patch of ground is cultivated, the rural area in which we did our research in primarily a fishing rather than an agrarian economy, which may have meant the issues of climate, which definitely impacts Malawi, was not mentioned.

# Basic needs

Under the attribute of basic needs, the participants talked about the things they considered to be the basic necessities for one to survive. The importance of being able to have basic needs was the same across all ages, genders as well as settings. The basic needs mentioned included food, shelter, clothes, water and electricity.

The participants were asked to describe their understanding of basic needs. Most of the participants had a similar understanding of what basic needs are with a few outliers. For the most part, the participants mentioned food, shelter, clothes, water and electricity. As mentioned earlier that there were some outliers, the participant below considered religion to be part of one’s basic needs above everything else:

I: What factors contribute to poor quality of your life? Maybe, things you would like to see change in your life? P: The things I would like to see change in my life is for me to be deeply rooted in a life of prayer and household basic needs for my everyday life. Things like foods, clothes, soap and salt. We usually want those things to be available at our household, since they are basic needs in a person’s life. I: You have talked about basic needs. What do you consider to be the basic necessities of a good life? P: For someone to have a good life? I: Yes. P: Firstly, involving prayer in everything. The first thing that a person need to do in order to have a good life is a life of prayer. Secondly, working very hard when undertaking a task you feel can make you get the things you lack. I: Things you lack, like what? P: Like basic needs. I mentioned about foods, soap, salt, clothes and dwelling place. Mw 22 Urban M 30

As for the participant below, she valued food so much that having a piece of land for subsistence farming was considered a basic need:

I: I asked questions about things you consider to be key things. According to your opinion, what things do you consider to be the basic necessities of a good life? What are the basic necessities of a good life? P: The first thing that is required for someone to have a good life is to have food at home. If we have food at home, we can have a good life. The second thing after having food, is to keep money for buying relish, soap, body lotion so that children should bathe and wash clothes. In so doing, we can have a good life. The third thing is to lease a land for growing crops because it helps us to have food at home after harvesting. So for someone to have a good life, she is supposed to have food at home. Mw 19 Urban F 45

Some of the participants mentioned financial security as a basic need:

P: A person need to have a home, a good house with solid foundation, enough food, tap water and electricity should be available. Above all, she should earn money through employment or engage in a big capital business that can provide for things needed at the household. In so doing, things can work well for someone. Mw 25 Urban F 63

For the participant below, in addition to everything else, being able to fulfil one’s responsibilities was considered to be a basic need:

What are the aspects of your life that contribute to poor quality of your life? Factors that contribute to poor quality of your life? P: Lack of basic needs. As I said, food should be available. I should have an opportunity of buying clothes. An opportunity or privilege of having a house, as I said. We pay water and electricity bills at home, so I need to manage all those things. I should also be able to assist my relatives. As I said, I have a disabled relative, so I need to manage all that. In so doing, it means I am having a quality life. Mw 26 Urban M 40

## Food as a basic need

Having enough food as a basic need was a common report among the participants. Their reason behind this was that one cannot survive without food:

Our bodies require food and we can’t stay alive without food. Mw 5 Rural F 40

I: We are about to conclude the interview. What things do you value in your life? P: The most valuable thing in our life here on earth, first it’s the food that we ingest in our bodies. Secondly, children are supposed to have accessible water. Mw 16 Rural M 41

Basic necessities for good life is for a person to get at lesat three meals per day, a person has to get at least three meals per day in the morning, afternoon and at night that way I see tat at lest its quality of life, one should get clothes and a good place to sleep that is quality of life, to be with good health, get education, get health services, water i mean those basic services. Tz 6 URBAN F 33

I: Apart from monetary issues. Having quality life does not simply mean having money. What are the other key aspects that are not financially related that contribute to your quality of life? P: Other aspects that contribute to my quality life. Whenever I do business, it also brings quality to my life because it helps me to raise money for assisting my children. In addition, availability of food at home also contribute to my quality of life. Mw 19 Urban F 45

## Clothes as a basic need

Clothes were mentioned as a basic need because they are a source of decency:

I: How about food, clothes are they important? P: Yes, clothing, housing, food are very important things I: Why do you think they are very important for good life? P: If you don't eat you will not live and if you live it will be by suffering, also clothing is important as you cannot walk without clothes. Tz 16 URBAN M 39

I: Are there things that are lacking in your life? Things that would have contributed to your quality of life, had it been that you had them? P: If someone doesn’t have support, she gets confused and engage in unwanted behaviors. I: What kind of support? P: Monetary support. Money is needed in every person’s life because without money a person can’t have life. I: Money assist in what way? What are the things that require money? P: To buy clothes and foods. Mw 23 Urban F 40

What are the aspects of your life that contribute to poor quality of your life? Factors that contribute to poor quality of your life? P: Lack of basic needs. As I said, food should be available. I should have an opportunity of buying clothes. An opportunity or privilege of having a house, as I said. We pay water and electricity bills at home, so I need to manage all those things. I should also be able to assist my relatives. As I said, I have a disabled relative, so I need to manage all that. In so doing, it means I am having a quality life. Mw 26 Urban M 40

## Shelter as a basic need

The participants mentioned having a decent place to stay as a basic need:

I: What do you think can contribute to your quality of life? P: The first thing is shelter when you get a good place to sleep it’s one thing. Tz 13 URBAN M 19

In term of your surroundings, like how you stay in your house and your standard of living. How does this affect your quality of life? P: I think to the quality of living, though as you can see this house is not fully completed. However, I thank God that I have been in tobacco industry throughout my working time, so I decided to build a house. We are staying peacefully, and I think that is part of life, we are comfortable. Sometimes we even get scared that why did we build this type of house. We knew that in the end, we might just stay the two of us with our grandchildren, so we needed to have such kind of a structure. So we are comfortable. Surprisingly, sometimes it happens that people who have ever come here, they ask if they can conduct certain events here. So, in reality, God is good and we are okay. Mw 27 Urban M 69

The participant below mentioned a house as being a source of financial security because he doesn’t worry about rent as well as in the future if his children decided to sell the house:

One of the opportunities is building a house like this one, because when we die, whether children will stay in it or sell it. Whatever they will decide to do with it, let them do with it. Opportunities that one. Even though, it’s a little bit at a standstill, but we have built a house and we are happy. When people come from my home village, they come in this house and sleep well. They find food. I can’t say that we sleep on an empty stomach. As I said, we grow crops and harvest enough crops for selling as well as consumption. Having a house is one of the highest opportunities in my life. At my age, at the moment, I don’t get salary and pension. I was working at a tobacco company, so I don’t get pension. They already gave us money. So, if it was not for putting up this structure, we should have been in the shit, but we are living properly. Of course, we are struggling to pay for electricity and water bills, but we don’t pay rent. Mw 27 Urban M 69

## Water as a basic need

Water was mentioned as a basic need for domestic use. The other thing the participants talked about were issues with access as well as availability of water:

I: Later? I want you to take to the time of sleeping P: You find my be if I am not tired I will take shower, if I am tired I may not shower till the next day or I may not shower that next day you know life in town because of the environment where I am living there is no water close, the source if water is far. Tz 12 URBAN M 30

I: What aspects of life that contributes to quality of life to be high? P: The first thing is water, hospital, school, what we get at home I: Like? P: Livestock that we keep at home I: How do they contribute to quality of life? P: Education opens mind to know that if i do this i will get that, the second is water without it life cannot continue, when you have food life cannot be poor, children that you have you get satisfied and see that they are your life. Tz 33 RURAL M 65

This issue of water has affected my life because water is vital, and water is life. I walk from here up to [name] School, so I feel like we are being mistreated. Mw 7 Rural F 60

## Electricity as a source of meeting basic needs

Electricity was a common topic among the urban participants. The participants talked about a number of ways by which electricity is a source of meeting basic needs. For example, the participant below describes how electricity is useful in the processing of food:

I: Is there anything that satisfies you in this village?

P: I am satisfied because there is no commotion, however, the bad thing about this village is that there is no electricity. So we always go to [name of other village] for everything.

I: How has unavailability of electricity affected your life?

P: It’s very difficult.

I: Difficult in what way? Can you explain?

P: Maize mills are faraway.

I: Maize mills are faraway. Where do you access it?

P: At [name of village]. I: So you carry the maize from here up to [name of other village]?

P: We carry them on our heads, as well as charging of phones. For me to leave here up to there. I sometimes miss some calls because I find it difficult to go and charge my phone at a faraway place. Mw 18 Urban F 30

The participant below mentions all the domestic uses of electricity:

I: What about in terms of electricity?

P: I don’t stay in an electrified house. We either use solar power or candles, but as of now, candles are expensive.

I: Are you satisfied that you use solar power or candles instead of electricity?

P: No, every person desires a good life. Electricity partly contributes to someone’s quality of life and improvement, so I am not satisfied, as of now. I desire to stay in an electrified house, but I can’t manage as of now because electricity is expensive.

I: How is electricity important to you?

P: Electricity simplifies things. At present, we are having problems in accessing charcoal for cooking because it contributes to deforestation. This is why the forestry department forbids that, so what will we use for cooking. Electricity is important because we use it for cooking, ironing clothes, lighting, radios and watching TV, whenever we have an opportunity. However, we fail to do that due to unavailability of electricity. Mw 20 Urban M 48

The participant below describes the use of electricity in the healthcare setting:

I: What is the importance of having electricity at your house?

P: Electricity is important because for children to study during the night, they need light. If I can compare to where we are coming from. There are other people who use traditional kerosene lamps [koloboyi] for lighting their houses. They live differently compared to people who dwell in electrified houses. Dwelling in an electrified house is a unique and valuable way of life. I feel electricity is important in a person’s life. When a sick person go to the hospital and is referred to ICU [Intensive Care Unit], there will be a need of electricity. So electricity is important. Mw 21 Urban F 58

# Achievement and personal development

Achievement and personal develop was important as aspects of being able to lead a good life or have good quality of life by some. Some participants were disappointed by not taking opportunities as they kept on living with parents that limited their exposure that would later be discovered after they leave home to start own business.

I: We are concluding. I was asking about opportunities. What are the opportunities that you value? Even though you lack certain things, there are certain things that you thank God for. P: As I said, in the past, I was staying with my parents. When I left my parents’ house and start living in my own house, I didn’t know how to run business because I was just fed by my parents. At the moment, I live here and I still decide to do certain things, as I am running my business. Mw 12 Rural F 29.

On the other hand, participants were proud of the achievements they have made as part of quality of life most of which are resulting in their personal struggle and resulting from bearing children that makes them to get out and work to earn for the children they have. They clearly stated to leaving parent’s houses to search for a better life.

P: Basically, I just decided that I shouldn’t continue living with my parents. After I gave birth and my child started schooling, I felt that if I continued to live with my parents, I wouldn’t do any wise thing. So I decided to leave so that I should live independently. I wanted to develop myself in many things. P: At present, my life is better because since I started doing business and doing shares, I have done certain things, as of now. Mw 12 Rural F 29.

On the same note, participants are concerned the way others look at them even if they have no money but they utilize the little present to keep themselves in good impression that is appreciated by the community around.

P: Sometimes they come to me and say that they admire the way I look after my body. I bathe, and even if I put on old clothes, I properly wash them so that they should look good, so they admire this. They say that when they enter inside this house, everything looks tidy. The tidiness of my house, without basing on financial status, so they admire me. My friends visit me and say such kind of things. Mw 19 Urban F 45.

Likewise, participants with no chronic illness from Tanzania and Malawi urban had disappointment to have not been able to properly care for their children better that is usually because of lack of money, dependence to the parents and single parenthood.

P: The other thing is about the future of my children. I contemplate on how their future will be. Every child who is born, mostly rely on her parents for assistance. I indeed have children, but I mostly rely on my parents, so what if my parents pass on. Mw 18 Urban F 30.

P: At times lacking school fees, at times a child needs something i fail to provide because i am unable to support because i am alone. Tz 1 URBAN F 45.

Not fulfilling plans for housing was another aspect that was contributing to poor quality of life. This was more common among male participants living in urban areas who had to pay rent, the amount that would have been used in other income generating activities.

P: Just being in your own house even if that day you have no feed you feel comfort, I have not got food but I am at my place there is no one to disturb me or ask me, I will start my life tomorrow. Tz 8 URBAN M 64.

Another disappointment that contributes to poor quality of life was not getting or providing good education and other needs to children. This was true among males and female participants with and without chronic illness from Tanzania and Malawi. Parents were not sure of the future of their children without education that they believe determines employment in future.

P: I have children, so I have given them a huge task. In future, they will have problems in finding a garden for growing crops. Unless, they become educated and stay in town. Mw 4 Rural M 67.

P: At times lacking school fees, at times a child needs something i fail to provide because i am unable to support because i am alone. Tz 1 URBAN F 45.

Furthermore some participants had own concerns for not going to school that contributes the current quality of life they have, which would have been different if they had received education as it would have determined to have good employment.

P: If i had gone to school you would have found me employed may be in the office, but because of not having education this is when you find that i see life may be is bad but its not bad what you get you thank God. Tz 9 URBAN M 56.

Participants showed regrets in life that contributes to current quality of life that includes time wastage during adolescence, not putting much efforts during school, these were considered as mistakes done that cannot be reversed but realized in the future life.

P: What i can say that sometimes i think of is my adolescence, at times I feel if I was to get back to adolescence there are things I wouldn’t do that I have done already, mistakes that have already been done, not only that but what pains more is education what pains me more is education that I would have put in more efforts more than I think now to make sure I get to a certain point and be somebody, they are things that I think of and feel that I lost so much. Tz 6 URBAN F 33.

However, fulfilling plans for housing was the aspect that participants with chronic illness from Tanzania and Malawi rural and urban enjoyed having accomplished in their lives. Having dreams and spend money to secure plots for housing making sure they enjoy life while still alive in own houses and to avoid suffering.

P: When I was still a boy, my goal was to have a brighter future. As I said, I was buying plots and building houses because I knew that l can either lose my life or carry on with it. If it happens that I am still alive, what will I do. I was doing that in order to have a good life when I am still alive. I thought about that when I was still a young man and that is what has made me to reach this point. I knew that I can either lose my life or carry on with it. I was planning for my future life and this is how far I have reached. Mw 17 Urban M 63

P: Without that house I will not be able to live I will be suffering a lot. Tz 9 URBAN M 56.

More so, quality of life was contributed by the way participants live with their children for both Tanzania and Malawi. They clearly showed being proud of their children by teaching them good conduct, respect to people as well as the way children listen and follow their teachings. Together with that, it was clear that bearing children increases being responsible when you earn you know there are children who have needs, this practice is different to people who have no children and therefore their expenditure being different.

P: The other thing that bring quality to my life is the way I live with my children. I counsel and teach them good conducts and the children are supposed to be obedient. If they don’t follow my advice, I become disappointed. They are supposed to follow my advice because I teach them how they can relate with people. If they meet with people, they are supposed to give respect even if they are not their relatives. Mw 19 Urban F 45

P: When you have a family you must know a child needs this and that because at first you had no child so even if you get money you will say i don't have a child what is the use of my money i can do this and that, but when you have a child your brain expands that i have to do one, two three. Tz 11 URBAN F 33.

Working hard stood out as a main source of achievement for both participants from Tanzania and Malawi, having that ability to get out, work and earn as well as working hard with a vision of getting somewhere in business as well as realizing that its working hard that will add value to quality of life.

P: What is contributing in the quality of life its that God has given me ability to get out and do such an activity and earn, so if I earn it means there is quality however much its hard. Tz 7 URBAN F 62.

P: If I can work hard in my business, it can take me somewhere. I need to do business with a vision of reaching a certain level, rather than just doing business for the sake of doing it. I have to weigh myself that what have I done this year. What were my plans. My plans were to accomplish certain things, so how did it go. Mw 24 Urban F 26.

Pride in educational achievement or being able to provide good education was another aspect found in this study. It was realized that taking children to school does not contribute to poor quality of life but rather their right and therefore a parent should fight to make sure they get education. More importantly it was important because children later help parents after they have got their employment.

P: Taking children to school does not reduce to quality of life but its a child's right to school even if you don't have you will fight to make sure, so there is nothing like poor quality or what, its a must. Tz 11 URBAN F 33.

P: It’s important because the children I look after also assist me. I am grateful for this. So looking after children is very important because I am being assisted by my children. Mw 2 Rural F 43.

# Attachment, Love and Friendship

Attachment was a theme running through the accounts of almost all participants. Participants talked about experiences of both positive and negative relationships, focusing largely on their own experiences, but including previous and current experiences. People spoke about their attachment, and love, of family, the role of friends and neighbours, and also that they enjoyed living peacefully with family, friends and neighbours. However, people also talked about negative experience of family, friends and neighbours which detracted from their quality of life.

## Attachment with family

### Valuing family in general

Men and women of all ages, and in both countries but especially in Tanzania, discussed the value that they attached family in general.

Some spoke simply about enjoying their company:

I: What do you value in your life? P: My marriage, children and my parents. Tz 32 RURAL F 45

I: What aspects do you value in life relatives. Tz 30 RURAL M 26

P: I love them [family] and they depend on me so I must value them more than anything. Tz 16 URBAN M 39

My relationship with my wife, kids, my parents and my parents-in-law. They shape us together. Mw 11 Rural M 34

By family, I mean my wife, children, younger brother as well as my one and only sister. I really enjoy with them. Mw 4 Rural M 67

For some, the love of family helps overcome other very difficult circumstances:

The basic thing is happiness and love in my life, it gives me a very good life even if I don't eat, when there is love at home, I see my life is just fine. Tz 3 URBAN F 64

### Parents

Men and women of all ages, and in both countries but especially in Tanzania, discussed the value that they attached family in general. Some especially valued their parents:

I: What do you enjoy? P: My family and parents in general - When you look at your parents they gave birth to you and took care of you till you reached age for school, after that may be you got employment or not, may be a man came and loved you it's because of your parents, you get married may be while at your husband's place you get employed that is parents fruits if not parents you would not get there that is why I am telling I like my parents so much, if I had enough ability for my house and my family I would not stop helping them, only that I base at home because the income is small. Tz 32 RURAL F 45

P: It’s the parents. When I was a child, the important thing in my life was my parents. Mw 30 Urban F 28

I value my mother so much I: Why your mother? P: She is my everything, she took care of me more than even my father. Tz 30 RURAL M 26

Sometimes parents are valued for the very practical help of helping with grandchildren

P: Firstly, it’s because I have both parents. I: Because you have both parents. P: This is the key thing. Had it been that it wasn’t like that, it would have been a huge problem. When going to work, I don’t leave my children with a house maid at home, they stay with my parents. This is why I even find an opportunity of going to do business and to my workplace. Mw 18 Urban F 30

### Spouse

Being close with a spouse was highly valued by some participants which could bring great comfort in working together and supporting one another to make important decisions or planning activities:

P: I will start with my family. My wife assists me in many things because we encourage each other in our physical and spiritual life. In life, we encounter challenges and other things. If I lack wisdom, we discuss on how to solve certain things and come up with a solution. When that problem is too big for us, we inform our relatives about that problem. If they are able to assist, they provide that assistance. If there are other people whom we fully trust and have good relationships with, we sometimes approach them and they assist us with ideas on how we can go through a certain thing. Mw 26 Urban M 40

I: What else do you value? P: My husband and we listen to one another there is no any problem, we decide together that is my happiness in my life Tz 23 RURAL F 44

P: Its listening to my wife we plan our activities, God helps us to continue with life a little better. Tz 34 RURAL M 53

Bringing up children together brought enjoyment:

I: And why do you enjoy having your wife? P: I enjoy because 'we help one another taking care of children. Tz 16 URBAN M 39

I love my wife because she has been able to give me those children. Tz 9 URBAN M 56

Some mention friendship as part of their relationship

P: There is good relationship because I treat my wife as my friend, so that is how we stay (participant laughing). I don’t treat her as my wife but friend. We live peacefully here in our everyday life, even with my children. Some of my children have gone to work, and I stay with some of them. So we live peacefully, without any problem. Mw 17 Urban M 63

And some retain elements of their relationship even if one is ill,

I: Tell me about your wife P: I value my wife, very bad that she had blood pressure and she cannot do anything but she wakes me up asking how I am doing we planning home issues then life goes on. Tz 20 RURAL M 77

Some simply valued having had a good relationship, with no disagreements within their marriage

Another thing that I enjoy, I am living good with my husband there is no any problem, so I see like life goes on well, there is no problems, no fight whatever is available is okay if we get its okay if we don't it’s okay. Tz 7 URBAN F 62

: I enjoy peace I have with my husband, he has never mistreated me it came a time people were talking bad but he said we are the ones married those who are talking let them do let's not listen to them, let's take care of our children if God blessed me with a wife who sees value in children who took care of young children I was left with so please lets fight for our life, I thank God we are safe. Tz 27 RURAL F 50

Those who have lost their spouse explain how difficult that is

P: My wife to die it’s what has changed my life abruptly, the income we used to get I don't get it on time I: What else? P: Children that I have at home they don't see their parent. Tz 33 RURAL M 65

### Children

Children were highly valued, again, especially mentioned by Tanzanians and some Malawians when asked what brought value to their lives,

I am also grateful because I have children. Mw 5 Rural F 40

At times when I am stressed when I see a child playing or jumping up down there my mind gets relief and when i hear that i am also called a mother i have children wow i feel happy. Tz 6 URBAN F 33

Grandchildren also brought enjoyment

I: What do you value in your life? P: My grandchild, he is like my last born because I am with him all the time, like now I am think of where will I find him (laughing) he has come from school already. Tz 23 RURAL F 44

P: enjoy because God has given me children that s one I: How do you enjoy having children? P: I enjoy because I see children as a comfort in my heart because I got married and stayed for two years without children I lived a misery life so when I got children I became happy, second I enjoy however much I have a lower life but I am at peace in my house so I even enjoy peace that I have, and we listened to one another as we plan, we share what we get its life that I enjoy, God has given me children. Tz 29 RURAL F 45

Children being obedient, or doing well, brought pleasure

I: What do you enjoy in your life P: It's having children that is what makes me enjoy I: How? P: Because when I have my children at home we eat together, cooperate you tell a child to do something they comply, that makes me happy Tz 26 RURAL M 55

P: I enjoy my children because I thank God on our plans with my husband God has helped, my children also are doing well there is no child that I am regretting that why is he like that, the good thing is the first child went to school she has finished she is a teacher and she is married, the second has finished God has helped he is a policeman, the third is at the college, I am with the fourth in form one, so what makes me happy is that if tell my children do this hey listen to me no one is rejecting so that also makes me happy. Tz 25 RURAL F 55

There is the time she makes me happy, my child makes me happy when she comes from school at times she has done good, brings the results it makes me happy, even if she doesn't do well you get mad but you come to a time and get satisfied because in life there is up and downs. Tz 10 URBAN F 50

Participants talked of being happy if their children were happy

I use most of my time taking care of my child, asking school questions, playing, I am very close to my child so that he gets used to me P: When I see happiness of my child I enjoy however much he has normal life. Tz 22 RURAL M 36

P: I enjoy my children that God gave me, it’s a blessing from God given to me like a gift so when I look at them I get happy. Tz 15 URBAN F 40

I am happy because if I buy my fish from the street and get back home and keep it in a flour tin when they come and open they will be like father has brought sauce that is happiness. Tz 19 RURAL M 35

And enjoying simply spending time with the children in their household, chatting or playing.

P: Chatting with my grandchildren. I entertain them and they ask me how I used to do certain things in the past, so it’s good. I: How do you feel when doing those things? P: I feel happy because I have managed to see my grandchild while I am still alive, and I chat with them. But there is someone who never reached this point. Mw 21 Urban F 58

I: What is it that you enjoy? P: I have a family, so I chat with my children. The children are healthy and not feeling unwell. Mw 13 Rural M 41

One young man living in difficult circumstances in the same room as hie mother and younger siblings managed to find pleasure in being with the children:

[His young siblings] make me enjoy because of what they do there is a time they make you laugh and make you enjoy being at home. Tz 13 URBAN M 19

Whilst a man whose wife died, who was living with HIV and found life very difficult had some pleasure with his children:

I am happy because I have these children. When they come back, I like to play with them, show me how they have played at school, how did they play football that gives me happiness on the side of children especially this young one, otherwise when I am at home alone I ask God to take me any time. Tz 19 RURAL M 35

### Why family were valued

Family were valued for a range of reasons. Some family members helped when ill:

My relatives properly assist me. When I suffer a lot, like during the time I told you, they come to care for me and look after me until I recover, it’s when they go back. At the moment, because of the time I was suffering, I stay with my mother. She came in July last year [2021] because of the problems that I have in my body. She is failing to go home because she want to make sure that I have fully recovered from all the things I was suffering from, it’s when she will go back home. Mw 28 Urban F 51

The general assistance, financial or otherwise that families can offer was highly valued

My family cares for me here and this is why I am like this. Mw 4 Rural M 67

What I value in my life. For me to be seen like this, it’s because of my brother who was residing in Area 49. He played a major role of assisting me after my husband died in 1997. So my brother who was residing in Area 49 played a major role. During the time my husband died, there was nothing happening here at home. For this place to have a fence or this house to be plastered with cement and look like this, it’s because of my brother who was residing in Area 49. The water and electricity that I have at my house is also because of my brother. He played a major role of assisting me as his sister because I didn’t have anything [poor]. He assisted me so that I should at least be better off. Mw 25 Urban F 63

I: How does your relationship with your relatives contribute to your quality of life? P: I tell my brothers whenever I lack something. I pay rentals on my own, but when I lack small things because my business didn’t go well, my relatives assist me. Mw 29 Urban F 33

I: What are the key aspects of your life that contribute to your quality of life? P: Key aspects of my life, it’s only my children. I don’t have anywhere else where I can depend on, it’s only my children. Two of my daughters were living in South Africa with their families. My other child works at the border. They clear vehicles before entering here in Malawi. My children are kind-hearted and God should continue blessing them. They assist me in looking after my grandchildren. On my own, I can’t manage. My husband stopped working long time ago in 2005. As you have seen, our house is not completed. We started living in this house when it was not completed in 2005. We live here because my children told us not to go to the village. They said that we might face problems with my grandchildren because they were very young. But they are grown up now, so it’s better off. Mw 32 Urban F 68

Relatives were seen as a more secure source of support than friends:

P: Okay, my friends assist me up to a certain extent, while my relatives assist me for a long time. For instance, if I can feel sick, my friends can assist me up to a certain extent, while my relatives can assist me the entire period. Mw 20 Urban M 48

And there is expectation, and pleasure in, mutual support and of children to help in a family’s chores:

I sorely rely on myself. My mother was the only child in her family. She doesn’t have a brother or sister. When my mother is sick, I assist her. When I feel sick, it’s my children and mother who assist me. My mother sit beside and tell her grandchildren [her children] to assist me. Mw 2 Rural F 43

An opportunity that I value a lot is that God has given me younger brothers, relatives, children and my wives. We assist each other on how we should live, so I value them. Mw 4 Rural M 67

And children are a source of assistance with household chores:

My children are still young, so they haven’t started assisting me. I tell them to accompany me to the garden, in order to assist me in cultivating. They also assist me in fetching water, that’s all. Mw 5 Rural F 40

Malawian participants spoke of the help or assistance they provided family members:

: My relatives assisting us? [No]. It’s us who assist our relatives. There is my in-law’s daughter. Her mother and father passed on, so their daughter is sick at the village in Balaka. So today I was saying that, had it been that there was someone going there, I would have sent her a bag of maize and some fabric. Her husband divorced her long time ago, so I would have taken some fabric and send her, so that she should wear them. I don’t know what she puts on. She got married, but her husband abandoned her due to sickness. So I said this today. It’s us who are better off. Mw 32 Urban F 68

However, every child pass through certain challenges and we need to collect them on things that they are going astray. In their lives, as well as things relating to their education, so those are physical issues. Even my relatives, I act as a counsellor to my younger and elder brothers and even my cousins. I am not boasting, but we people have different gifts. On that part, they regard me as someone having a gift in advising them, so that when things go wrong, we should find the right way. Mw 26 Urban M 40

I: Do you enjoy sharing money with your relatives? P: Yes. I really enjoy because they are my relatives. I: You don’t get annoyed with this? P: Yes. I: Do you feel it’s beneficial for you to assist each other with your relatives? P: There is a benefit. If I can face problems tomorrow, it’s them who can assist me. Mw 15 Rural M 26

He doesn’t walk, but we walk. So when he feel sick, I go to cheer him up. When I prepare food, I go to give it to him. Mw 7 Rural F 60

Two younger adult Malawian participants spoke of valuing the advice their older relatives could offer:

P: Things that support my life. I am really grateful to the people I stay with because most of the times, they give me advice or guidelines that assist me in certain things, in order for my life to improve. In life, there are certain things that I can’t know if I am doing them in a proper way or in a certain way. With the guidelines that they give me, they assist me to see something. I feel it’s important to follow what parents or people whom I stay with advise or enlighten me. Even people who are close to me, because there are certain people who naturally like advising people. So such things encourage me and help to improve my life. In my life, I like people who tell me the truth rather than telling me lies. Even if that truth can be painful, it can still help me. Mw 24 Urban F 26

I: Do people sometimes influence or affect your life, not just with material things, that’s what I mean. P: You mean like giving advice? I: Yes, things like that. P: Yes, they advise me. Mw 30 Urban F 28

And one middle aged participant greatly valued her mother’s teaching, including about traditional cultural practices

P: The other things that bring quality to my life is about my parents. As I said, my father passed on and my mother solely rely on me for everything, even though I am poor. When my mother comes, we chat with her and she also teach us some of the things that they used to do, when we were still young. As of now, she also tells us some of the cultural practices. So it thrills my soul because I know that she is teaching us how to relate with people, because interacting with people is difficult. She teaches us that we are supposed to study people and what they like and in that way we can understand each other. If we don’t know each other well, we can’t understand each other. So, it thrills my soul when she gives me such counsel and I feel it’s important to have elderly people in our midst. Mw 19 Urban F 45

Finally, three participants (two Malawians and a Tanzanian) mentioned that they enjoyed visiting wider family members

I: Do you sometimes go somewhere? P: I go to town. I like going to my brother’s office. I also visit my aunt in [name of town] and my other relatives. Mw 29 Urban F 33

P: I enjoy remaining at home to rest, to be with my wife and children and my relatives are a little far but i visit them sometimes Tz 34 RURAL M 53

I don’t have friends whom we pay a visit to each other. I just visit my relatives. Mw 6 Rural F 27

And one that even though visiting family was hard, they made an effort to attend important events with family, like funerals

I: What are the situations that prompts you to pay a visit to each other? P: Whenever there is a funeral. We don’t regularly pay a visit to each other due to lack of transport. Whenever there is a funeral, we try to source money so that we can attend the burial ceremony. So we meet each other in such way. Mw 19 Urban F 45

## Attachment with friends and neighbours

### Friends

When prompted, participants in both countries, both sexes and all ages mentioned their friends as a source of value in their lives – although more Malawians talked about friends than Tanzanians. Participants explained that they enjoyed spending time with friends chatting:

Yes, I go to chat with my friends. Mw 8 Rural M 73

I: What else do you enjoy? P: Chatting with my friends. Mw 15 Rural M 26

P: I can’t solely depend on myself, so I enjoy staying in groups with my friends, so that we can know each other. During those interactions, we sometimes tell each other to do a certain income generating activity. Mw 23 Urban F 40

P: I have a friend whom I love from deep down my heart. I have a friend, but that friend also has another friend. I can comfortably discuss my personal things with him, but he can in turn tell his other friend those things. But I properly chat with my friend. Mw 16 Rural M 41

And participants mentioned enjoying visiting friends:

You asked me about my friends. There is good relationship because we pay a visit to each other. We don’t give each other things. They can give me things, but I don’t have things that I can give them. Mw 4 Rural M 67

I properly relate with them [friends]. They come here at home, and I also go to visit them at their homes. Even here at home, they know that they are my friends. Mw 3 Rural M 25

[I enjoy] visiting friends, may be getting a drink but if I don't have that may be [if I get money] I buy soda to drink with my children at home. Tz 1 URBAN F 45

One person talked of not needing many friends:

I: How about friends? P: When you get one or two friends that you are with in happiness and at sad times that is enough that is yours. Apart from that, no the whole village are not your friends. Tz 23 RURAL F 44

And another of moving on from having close friends as one aged and got busier:

As regards to my friends. As I said, one of my close friends is now in [name of city]. I have not really gone to [name of city] to go and chat. As I said, since I came here in September I have been involved in all these issues of land disputes, so I really didn’t have time to move around. /////. But if he comes to Lilongwe here, he comes and pass by. Even if he doesn’t pass by, at least we make calls and talk to each other. This is the same with my other friends. For others we talk through WhatsApp and all those kind of things. Since my friends are working, sometimes it’s also difficult for them to create time for that. So I have to accept because that’s exactly what work is all about. Mw 31 Urban M 49

Whilst one talked of being very careful to be sure friendships would lead to spiritual enhancement:

Apart from that, I really want to properly chat with my friends and that my friends shouldn’t contribute to my sadness. So it just depend on their spirituality, how they were born and bad behaviour as a result of what is happening nowadays. I really want everything about my physical life to be within the limits. Mw 26 Urban M 40

For some, friends were a source of social capital and their relationship was mutually beneficial:

[having friends] assists me in many ways because it’s like a social capital. For example, I have a cassava garden. When I harvest the cassava, I don’t sell it at the market. I use social capital. I just plan that I will sell my cassava in a certain area for two days and since I have friends in that area, it helps me to promote my business. People support me because of the way I chat with them. For instance, if I properly chat with you, then I bring sweet cassava to you and you taste it, you can still decide to buy it even if you don’t like cassava. So social capital assist me in many ways and it helps me to move forward. When someone buys my farm produce, it means he is supporting me. Interaction helps me to move forward. Mw 24 Urban F 26

An electrician explained how his social network of friends helped him get work as an electrician:

I: Are there some opportunities that you find because of someone? P: Yes, it happens because of my work. People say that you want an electrician, he is there. He is our neighbour or friend. So I am able to benefit through such kind of people. Mw 34 Urban M 30

Others talked of the mutual assistance that can be helpful with friendships:

If my fellow church member has a problem or is sick, I need also to rush there. In so doing, we build up our relationship and they even say that we shouldn’t forget those people. So this is how it’s supposed to be. Mw 27 Urban M 69

I: How about relationship with friends, family P: That is good life you visit a friend and you assist one another when you leave one another life becomes poor that is why you find a neighbour and relatives cooperate and assist each other. Tz 33 RURAL M 65

I: When you have problems, do you ask your friends for support? P: Yes. I: When they also have problems and have come to ask for support from you here, do you give them, when you have that thing? P: Yes. Mw 14 Rural F

And of helping friends as help is needed:

That is a daily routine, if you get something [earn some money] and your colleagues [friends] don't have you have to take care of them for them to live that day. Tz 4 URBAN M 23

And friends can be useful if one needs some help oneself:

I: Friends who are married like you are? P: They are there we normally tell each other about [the availability of] work. I go for them as a friend asking for any small support for my life. Tz 34 RURAL M 53

Sometimes my friend (name withheld) buys electricity units for me. Mw 9 Rural F 58

On participant spoke of being able to ask for help from friends but not neighbours:

I: If your lack something, does your neighbours or friend assist you? P: Not my neighbours. I: You talked about your friends. P: Maybe my friends. Some of my friends assist me when I lack something. Mw 30 Urban F 28

Friends were helpful even if they could not provide material assistance – they were helpful to make one feel better in difficult circumstances. One older woman, who was living in poverty with HIV explained the importance of her friends:

I only have one neighbour who is also my friend I just visit her when I am not well, especially when am stress, I go there we sit and chart and later when am ok I go back home and rest. That is a friend I may say I have. Tz 18 RURAL F 62

I: What makes you happy you happy in your life or what do you enjoy? P: Happiness is living at peace and love at my own home there is no happiness more than that. But when am annoyed I use to go to my friends who are good to me and we have a time to chart and thereafter I return in my home and sleep. Tz 18 RURAL F 62

And others were grateful to friends for visiting:

My friends come to cheer me up when I feel sick. When I am having problems, I don’t receive support from somewhere. My children who are married sometimes support me, but I don’t have relatives who can assist me. Since my father died, I am without help. Mw 2 Rural F 43

They visit me. I feel very happy when my fellow church members come, even though they apologize for not bringing anything. They say that they don’t have money. I tell them that it’s okay, they have done well by visiting me and giving me encouraging words. So I feel happy. Mw 1 Rural M 80

Friends were helpful not only for practical and emotional assistance but also for advice and information:

I: How do your friends or neighbours contribute to your quality of life? P: They assist me by enlightening me about other remedies like herbs. They tell me to try getting herbs somewhere because they also help. Like from Maranatha, Teras and others. They encourage me to try different remedies. When I try those things, I find that sometimes they work, sometimes not because it depends on the blood in our bodies. Our bodies differ, but many of them tell me to try different remedies. Mw 28 Urban F 51

I: For example on things like, what important things that you share with friends? P: being with friends it's just stories, to advise one another things like that. Tz 30 RURAL M 26

Sometimes I personally make the decision, but as you have said, it depends. If I want to make a huge decision, I sometimes ask for guidance from my female neighbour called (name withheld). I tell her that I am having problems in dealing with a certain issue, so what can I do, so she assists me or gives her views. The counsel that she gives is constructive not destructive. I also scrutinize the suggestion made by my friends, if it’s sensible or not. If it’s sensible, I then put it into practice. Mw 19 Urban F 45

Having friends is important because when I face problems, they can assist me with good advice. Even if, they can’t provide money, those views can assist me in many ways. I can have money, but my friend can teach me that I didn’t properly use the money, so I should do this and that. That can assist me in future. This is the importance of having a good friend. Mw 13 Rural M 41

My friends are important to my life because they encourage me in many things. When I have made an error, they tell me that I was supposed to do it in a certain way, so that it can work well. We teach each other many things. If they lack knowledge about certain things, they also call me and explain that to me. I tell them that they shouldn’t get worried because this is the current situation in the world. Mw 5 Rural F 40

Yes, when I am having a lot of difficulties in making decision, I consult my friend. I tell her that I want to do this, so how can I do it. If she knows that thing, she tell me that I should just do in a certain way. Mw 10 Rural F 50

### Neighbours

One Malawian participant enjoyed where they lived and asked about friends, said that neighbours were their friends – important for chatting to:

I: What about your friends? P: Those whom I chat with? I: Yes. P: We are surrounded by our friends here. Mw 32 Urban F 68

Both Malawian and Tanzanian participants, men and women, were pleased they had a good relationship with their neighbours, expressed as an ability to have a good, or proper, chat

I: What about your friends and neighbours? How does your relationship with them contribute to your quality of life? P: There is a good relationship and I really feel good. We came to live here, just after this neighbour settled here. This neighbour settled here long time ago, but there is a very good relationship. They are people whom I trust. Mw 32 Urban F 68

P: I don't have children, I don't have a husband people normally visit me we sit there and talk I: So you are good with neighbours P: So much, even if I am going somewhere I will tell them if I come late you will find they have come in and switched on the light and closed the door. Tz 31 RURAL F 57

P: My neighbours too we are good they are not bad to me we talk well. Tz 34 RURAL M 53

I: How is your relationship with your neighbours? P: We properly chat. Mw 15 Rural M 26

There is good relationship and I chat with them. Mw 2 Rural F 43

Sometimes our neighbours come to chat here at home. Mw 3 Rural M 25

Even if they were too busy to spend too much time with them

I: How is your relationship with your friends and neighbours? P: I can’t properly explain because I came here recently. However, I feel there is good relationship because we greet and talk to each other. We cheer each other up whenever one of us is sick. But, I can’t just be spending time with my friends every time. Mw 21 Urban F 58

I: Do you have a good relationship with your friends? P: Yes. I: If you have a problem or you

Although one said that although the relationship with neighbours was not bad, they were not close and suggesting that in earlier times neighbours would have been closer to one another.

According to my observation, we have moved a little bit in terms of the traditional Malawian culture that we used to have. So I can’t really say that we have got extreme relationship with my neighbours, but I don’t also have bad relationship with my neighbours. You get my point. What I am trying to put across is, if I say we have extreme good relationship, it’s something like, for example, I would go there to have a chat with him and all those kind of things. Maybe, he or her wife would come to have a chat with us, but we don’t do that. But at the same time, if there is anything, we are able to call each other and talk. We tell each other that my neighbour this is what has happened or ask each other that how is it going there and other things. I have their phone numbers, they have my phone number and we talk to each other when there is a need. But I don’t go to chat at my neighbours house. I need to be very truthful. Mw 31 Urban M 49

AS with friends, neighbours were valued because of the mutual assistance they could provide, or try to provide even if they lack financial resources to help much.

I: What about neighbours or fellow villagers? P: I live peacefully with my neighbours. As human beings, sometimes you find that some of our neighbours wish us bad things, but I live peacefully with my neighbours. I: Do you assist one another whenever one of you is having a minor or major problem? P: Yes, we assist each other. We have formed some small groups that we call Chineba. I have joined two Chineba groups. When I went to construct the sepulcher, one of the Chineba group followed me there. My fellow members of village bank also visited me. So I was pleased with this and this is the way to go. Mw 19 Urban F 45

I: Do you mostly rely on them whenever you have a problem? P: Yes, like those that stay here. We pay a visit to each other whenever one of us has a problem. When I am admitted at the hospital, they come to cheer me up. Some of them visited me here at home, after being discharged from the hospital. They said that they failed to come to the hospital due to lack of transport money, so this is how it happens. Mw 19 Urban F 45

I: If a certain thing happen here in the village, do you come together? P: We come together. When I feel sick, they come to cheer me up. If they also feel sick, I also go to cheer them up. Mw 12 Rural F 29

I: Friends, neighbours P: We help one another with neighbours if you don't have. Tz 15 URBAN F 40

One talked of the help he got from a neighbour when he needed food, although he did not feel good about having to ask.

I: How is your relationship with neighbours? P: There is one neighbour who is a sub village chair where I live, is the one who keeps me at times you feel bad going at his place because today he gives you a tin of maize, the next day going there again and again for begging I feel bad. Tz 19 RURAL M 35

Like friends, neighbours could be a good source of advice

There is good relationship. I go together with them when ordering things. I ask them how they do certain things and teach each other things. Mw 2 Rural F 43

One Tanzanian participant, who was community worker, spoke about how much they wanted to help their neighbours even though they did not have much to give.

P: When you have a neighbour or relatives who is poor, when you want to help you find your income is also small or even if it's small you have to give a little for him/her progress a little. Tz 32 RURAL F 45

And she went on to say that it made her feel good to be an important member of her community.

I: What contributes quality to your life? P: My children I: Tell me like four, five things P: Children, my work, how I believe in myself, the way the community takes me, for sure the community takes me good and they still need me I: The community needs you? RL The way I have lived 16 years in the community around for sure they need me be based on how I work and the way I live with them I: How do you live with them? P: Just love I: What kind of love? P: May be as I provide services they get happy. Tz 32 RURAL F 45

## Enjoying living peacefully with family, friends, or neighbours

When asked what they valued in life, or what opportunities they had some (both Malawian and Tanzanian, men and women, in both settings) spoke about enjoying living peacefully with family, friends or neighbours. They spoke in general terms, about peace being important:

I: What opportunities **or freedoms do you value? P: Okay, the freedoms that I mostly value are peace and love amongst people. If there is love amongst people, many things can’t happen. The bible says love your neighbour as yourself, so if I love my friend, I can’t offend him. There should be love amongst us because God is love. If I have love, it becomes difficult to offend someone. So I mostly value love and peace. Loving other people. Those are the things I mostly value. If I love my friend, I can’t think evil things against him. I also respect every person, whether young or old. They say that if I want someone to respect me, I need to be the first to give respect. I know that if I give respect to every person, people will also give me respect because I give them respect. I also value discipline a lot. Mw 24 Urban F 26**

**I: What freedom or opportunities do you value in life? P: Peace is everything in life, when you have life discipline because my teacher told me that when you have freedom to do anything to developed your life you must be interested also to have faith that you can do something also religion not to forget your God on everything you do, you will achieve. Tz 22 RURAL M 36**

**P: A family issue I think comes to the things that can improve life, when you are in a family with peace and love you can improve but when you are in a family that there is no understanding and fights it can be a challenge to quality of life. Tz 30 RURAL M 26**

**I: Other things that brings your quality of life? P: Its loving one another patience, its what brings quality of life**

**I: What else do you** value? P: Living good with people even the bad ones surrounding you its just teaching people to love one another. Tz 28 RURAL M 67

And also in specific terms, about enjoying living peacefully with their own family, friends and neighbours.

I: What about your relatives and friends? P: I live peacefully with my friends and relatives. Mw 17 Urban M 63

I: Okay, what contributes value to your life? P: The first thing is loving my family and the second is working because God had not denied me to work, I still have strength to do small activities I have told you and to loving all people and self-respect with my neighbours, that is what I see contributes to quality. Tz 14 URBAN F 63

P: I do enjoy living peace and love with my friends and relatives and other relatives. Tz 18 RURAL F 62

I: Okay, what do you enjoy in your life? P: What I enjoy in life is peace I: How does peace make you enjoy? P: Because when you live in peace you find yourself happy all the time that is what I like in life. Tz 13 URBAN M 19

I also enjoy staying here at home, without quarrelling with anyone. I really enjoy this. I just stay from sunrise up to sunset, without quarrelling with anyone. I just live peacefully here. Mw 1 Rural M 80

I am happy with my children, the way I live with my children in peace. Tz 3 URBAN F 64

The relationship is good i don't have fights with neighbours or relatives, mistakes are human I cannot deny that but when you realise a mistake go to that neighbour and tell him/her that there is this mistake that has happened but let’s leave it and forgive one another. Tz 9 URBAN M 56

## Negative experiences

Reinforcing the importance of attachment with others, some spoke of negative experiences of both family, friends and neighbours.

### Negative experience of family

In speaking about what brough a poor quality of life, some in both Malawi and Tanzania spoke about difficulties with their families. Women and one man spoke about difficulties with their husbands. Some were divorced or separated but were bitter about being left in sole responsibility for their children:

P: I went to the court after we separated. The court asked me that what properties do you have, so I explained the things that we had. But since I was very angry, I told them that I don’t need anything. What I needed was peace of mind because I was ill-treated at the marriage. What I wanted was my child and my life. So what I wanted in my life happened because the divorce happened and I took the child. He was from Northern region, so the child belongs to the father’s side [patrilineal], but according to how we separated and what I explained about my child, the court authorized me to take the child. As regards to financial issues, since he married another wife, I didn’t want to argue with him on financial issues, at a time when he already married another wife who was staying in my house. For me to say that I need a house, I was thinking that I would have ended up being bewitched. I would have lost my life and that of my child because of a house and car. So I decided to leave them. I told them that what I wanted was my child, so I was given the child and I left the possessions. Mw 29 Urban F 33

I: Are you satisfied with the support that the father provide to his child? P: No. I: What would you have loved to happen? P: It’s just the same as staying alone. I should be doing business in order to look after my child. I: How has this affected your life? The father of your child failing to provide the usual support, how has this affected your life? P: He says that he doesn’t have money. I: Are you satisfied with the response that he gives you? P: No. I: The way you know him, does he really lack money? P: I don’t know the money that he has in his pocket. I: How about the way he looks? P: I don’t know because husbands who are polygamist can’t be trusted. Mw 14 Rural F

I: Did your former husband left for South Africa after you got married? P: I gave birth to these two children when I was still staying with him. Later on, we agreed in our marriage that it’s better for him to go to work in South Africa so that our lives can improve. We should stop relying on our parents and become independent. I: How did it go after he went to South Africa? P: When he went to South Africa, he changed his mind and married another wife there. So I don’t receive any support from him. I: You also have another husband here. Do you have a child with your current husband? P: No. I: So you want your former husband’s children, who reside in South Africa, to be given adequate care? P: Yes, he should be assisting his children. He should be providing things that the children need for their education and clothes. I: They are currently lacking clothes? P: Yes. Mw 18 Urban F 30

I: The issue of her father supporting her occasionally, how does it affect you? P: I am affected, but there is nothing that I can do. I can’t force him because he doesn’t want to properly support the child. This is why I do it on my own, I can’t tussle with him. I: You have just accepted this? P: Yes. Mw 12 Rural F 29

I: What else contribute to poor quality of your life, apart from what you have said? P: The other thing that contribute to poor quality of my life is that I have a child but I look after her on my own. Had it been that her father was properly supporting her, maybe I would have been quickly solving some of these other problems. I: Do you have plans of reconciling with the father of your child? P: No. Mw 12 Rural F 29

In general, one person explained that poor quality of life can be caused by a husband who drinks a lot and uses a family’s income for himself:

I: What aspects contributes to poor quality of life? P: It can be poor because of conflicts because everyone does what he/she wants, everyone uses economy/earnings as they want either for profit or loss and you cannot plan any valuable thing you cannot plan to produce, one might want to change a place to live to avoid conflicts and one will fall in alcohol, for example many men get in alcoholism and spend the time there to avoid thoughts so that because there is no way and staying there doesn't mean he is just talking he will drink as well and that means that is money used and not alone he will buy for the other, if a woman sees that she will stop providing and life will be poor and the family will be affected, children will not be happy at school even livestock get affected if it's a cow will reduce on the level of milk because they will be not eating well, so the first thing in a family is understanding one another. Tz 29 RURAL F 45

Whilst another finds herself in conflict with her husband since she lost the intimacy of sexual relations with her husband after the menopause:

I: I would also like to know what important things in life that have changed are. P: Things that have changed, me and my life age has increased let's say may be there are things I used to do but now I see like for example in marriage I was living with my husband at peace but after age has increased and having children, when I stopped getting in menstruation that condition of feeling being with my husband reduced I see like that peace, that love has changed other things we were sharing I have to do on my own, by then I was strong but some other things I don't get success because at first we were sharing like we have got this lets do this if its school fees, but these days may be he sees may be there is no need to be together (sleep) if I want to bring it back I see it doesn't work out. Tz 25 RURAL F 55

One woman described at length the problems she was facing with her husband’s family, and subsequently with her husband

I: We will now discuss about factors that contribute to poor quality of your life. What factors contribute to poor quality of your life? P: Factors that contribute to poor quality of my life. ////// I was facing huge problems with what was happening to me. I got married in 2000 and since that time, we have been staying peacefully and our marriage was a role model. In 2013, it’s when my husband’s relatives started causing problems. They were saying that they don’t want me and their relative [her husband] should marry another wife because they marry women from their home village. I am a Chewa from Lumbadzi and my husband is from Ntcheu. Love is difficult because people sometimes fall in love wherever they meet and it’s not based on where someone comes from. They said that at their home village that’s how it happens, but I got married with him because we loved each other. However, in the course of our marriage it’s when I started experiencing this. His relatives started tormenting me and telling me to leave this place. We were also staying at our own plot before coming here. We bought this plot and started staying here in 2006, thereafter, it’s when the quarrels started. They were telling me to leave because they don’t want me, so there were arguments. My husband started following what his relatives were saying and the love that he was showing to me at first changed. So I was concerned because of the things that I was passing through in my life. Later on, my husband married another wife and he started beating me. Things were not okay, he was angry with me and we were disagreeing. Mw 19 Urban F 45

And one man spoke of his wife’s annoyance with him for coming home late as being difficult to manage.

I: We will now discuss about factors that contribute to poor quality of your life. What are the factors that contribute to poor quality of your life? P: The thing that contribute to poor quality of my life sometimes is arriving late at home. When I come back from the places I talked about, I meet my wife at home. Instead of her welcoming me, as her husband, despite that, I have arrived late, you find that she starts shouting at me. I know that I have offended her by coming back late, but we end up arguing because of the way she talk to me. So if my body was at a certain point, it reverts to where I was. . I: You mean having some disagreements at home? P: Yes. Mw 16 Rural M 41

Others spoke of disappointments with children or grandchildren, for example, when an older child starts drinking or taking drugs:

I: Apart from that what do you think contributes to poor quality of life? P: Not getting your right, you want to do this you fail somewhere I: Can you explain a little P: For example you expected my child to study and it reaches a time he/she rejects so when he/she comes back home he is dependent, what he does is not good at times he may become a thief or a use drugs, he doesn't bring anything home you keep on thinking what you get is used with no profit, also climate contributes to poor quality because you expected to plant and rain doesn't come, and others. Tz 25 RURAL F 55

P: The way I stay with my children this time is difficult. Children think that drinking beer is what makes them enjoy. When they drink beer, they come back and start shouting at me. If they are not enjoying the relish I have prepared that day because of the beer they have drunk. Perhaps, they wanted to eat meat. So they make a lot of noise. Since I am hypertensive, this causes my blood pressure to rise, so can I have quality life in this regard. I can’t have quality life, but poor quality of life because of the challenges I am experiencing. So that’s about my children. Mw 21 Urban F 58

I: What things would you like to see change? Things which can lead to an improvement in your quality of life if they can change? P: Things that can improve my quality of life, if they can change. Firstly, if there can be a spirit of togetherness with my children and they shouldn’t be troubling me. As you know, children nowadays sometimes engage in bad habits like drinking beer, so I don’t like that. I want us to be living in peace, harmony and I shouldn’t be distressed. Mw 19 Urban F 45

And others had difficult relationships with other relatives – often in-laws but also siblings and their families – seemingly because of the stigma HIV brought on participants

I: How about relationship? P: Yes they are changes with relatives after knowing i am sick she started rumours on me. Tz 34 RURAL M 53

? P: In this community, all my relatives have children except me I don’t have a child. But if I see the way they live with me, they despise me they don’t value me at all as their relative, I am saying this and God is hearing me. My relatives sometime they blame me that, I curse them but no I am just speaking that because they don’t value me at all, They sometime say in front of me that they don’t beg from people (participant sometimes asks her brother and family for food or money because she is frequently hungry), I usually don’t reply I just keep quiet. I decide not to visit them and I just stay in my home. They just speak these words because I don’t have a husband or a child, I don have any help even from them. Tz 18 RURAL F 62

P: The other thing that contribute to poor quality of my life is when someone has spoken unkind words to me. The other things that hugely concerns me is that, as I said, my mother solely rely on me as her elder child. I have my brother who is troublesome, so whenever my mother calls me to inform me how the situation is there, it contributes to poor quality of my life because he is a male child who was supposed to be assisting my mother because my mother is growing old. So when I hear such things, it contribute to poor quality of my life. Mw 19 Urban F 45

I: What makes your Qol poor? P: What makes my life poor is this my brother who isolate me. My father was a business man. Before I get marry my father use to love me more that all his children because I was the last born. What makes me unhappy is that why should my brother when he found out that I have got a problem he got bad words on me and my family…. [Cry] Nobody write God and ask to have Malaria, it happens on a bad luck. And the way he use to harass me I cannot figure out who brought this disease [HIV] at home between myself and my wife. Basically he was supposed to speak to me nicely like the family the way we was when we were young. Tz 19 RURAL M 35

### Negative experiences of friends and neighbours

Asked specifically about how friends and neighbours helped their quality of life, some explained that they did not, and that relationships were challenging:

I: How about relationship with neighbours? P: On the side of neighbours there is a challenge, there are some whom you can be with all the times, there are others who wish to see you failing, not all are good they have many challenges I mean neighbours who are relatives but those from other regions have no problem but because we are used like here is at aunt's place, uncle, grandfather so they know your life the way it is. Others can get happy but many get annoyed with your life, or this wants to be higher than me. Tz 29 RURAL F 45

Or that when relationships are bad and neighbours won’t ‘properly chat’ it makes one feel bad:

I: What else contribute to poor quality of your life? P: The second thing is about how we respond and talk to each other, without regarding the presence of people. When I was in good mood and we were properly talking to each other at first, but things turn up the wrong way, I don’t feel happy. I: You mean how you talk to each other with your wife or other people? P: With other people. Mw 16 Rural M 41

One older man had a particularly poor relationship with a neighbour. He went on to explain a little more that the problem was due to a dispute about a building and that he thought the neighbour had bewitched him because of that.

The problems here in the village is that people are jealous. This is what creates problems for me and makes me feel sick. Others who consulted witchdoctors say that people have bewitched me due to jealousy. In the past, I was just okay. So all these things create problems for me. Mw 1 Rural M 80

At first, we used to pay a visit to each other. Like that neighbour, it’s me who gave him a place to build the house because he was my co-parent-in-law, but has risen against me. He said that I should remove this shelter because it’s close to his place, but it’s me who gave him that plot. I invited the chief so that we can discuss this. So this kind of behaviour negatively affect my life. When I go somewhere, I hear that I have a land wrangle with my neighbour. Mw 1 Rural M 80

There is good relationship with my relatives, but there are some whom we disagree because of their witchcraft practices. Other people revolted against him, so it was a difficult situation. When they visited a witchdoctor it’s when they were told that I was bewitched together with my younger brother’s son, so people revolted against him. We don’t have a good relationship with them, we stay with them in a difficult way. At present, the government doesn’t legalize witchcraft, so we have just left it like that. However, the truth of the matter is that witchcraft is real. Mw 1 Rural M 80

Others did not have a poor relationship as such, but recognised the limits of friendship – the friends and neighbours could not assist them

I: What about your friends or neighbours? How does your relationship with them contribute to your quality of life? P: My friends and neighbours, forget about it. They don’t assist me. I: Even your friends? P: Yes. I: You don’t have a friend whom you rely on, who can assist you if you can have a problem? P: Yes, not on these issues. I personally deal with them on my own. Mw 33 Urban M 68

My neighbors are not caring. Even if, I feel sick, they don’t go to cheer me up. I just stay with them because they are my neighbours. Mw 2 Rural F 43

My friends whom I chat with also lack money, so they can’t assist me in my life. Mw 3 Rural M 25

Even if, they can have money, they can’t give me. Mw 8 Rural M 73

And there were limits to friendships

I: What about your friends or neighbours? How does your relationship with them contribute to your quality of life? P: My friends and neighbours, forget about it. They don’t assist me. I: Even your friends? P: Yes. I: You don’t have a friend whom you rely on, who can assist you if you can have a problem? P: Yes, not on these issues. I personally deal with them on my own. Mw 33 Urban M 68

Some actively avoided friends because they were a bad influence,

I: How about friends? P: If you have drunkard friends you will get into that, like I have older friends who have positive contributions on what to do. Tz 30 RURAL M 26

I: What contributes to poor quality of life? P: Cooperating with friends who have no stand, they waste your time they don't advise you good things they don't tell you what to its only words to ruin your life to be poor. Tz 34 RURAL M 53

And another was frustrated because his housemates would smoke drugs all day and not contribute to the household food supply

You find that you go for work and find others uses marijuana and when you get back home they tell you no money and while they were the one's taking drugs, if you ask that we have been working together where has the money gone, they tell you my money is finished i have no money now. Tz 4 URBAN M 23

# Participation in community activities

Another important attribute mentioned by the participants as a determinant of good quality of life was participation in community activities. The participants talked about both the positive aspects as well as negative aspects of community. From the data, this attribute was important across all the three age groups, in both urban and rural settings and in both genders.

## Taking part in activity brings cooperation and support

One of the positive aspects brought up by the participants was the participation in community activities brings cooperation and support. From the two accounts below, we can see that the common community activity most participants associated with cooperation and support was the church. The participants talked about receiving support in times of sickness as well as lack:

I: How does your spirituality and church contribute to your quality of life? P: Our church support us reliably. We were members and church elders. Our church support us. We relate well with our fellow church members. If I can tell them now that I am not feeling well, you find that the women can come to cheer me up. Attending church services is important. Mw 32 Urban F 68

My church supports me. When I sometimes lack, they bring things and give me here. Mw 9 Rural F 58

For the Tanzanian female below, her reason for taking part in community activities is so she can also receive some form of support in the future in case she is also faced with some disaster.

I: Communities have things like disasters, do you attend? P: I do attend, I cannot miss that if I leave how about me tomorrow? Tz 18 RURAL F 62

As for the Tanzanian male below, he considers community gatherings as a source of support as well as knowledge, which in turn improve one’s health and eventually, quality of life.

P: Good health can improve quality of life, education can improve my quality of life also gatherings being with others I: Can you explain a little about being with others? P: For example I am with a certain group and one gets a problem, we must follow up closely and it helps that person to feel good. Tz 24 RURAL M 18

## Taking part brings enjoyment

Some of the participants reported feeling a sense of enjoyment from taking part in certain community activities. From the two accounts below, we again see the significance of the church as taking part in church activities is described as a source of joy for some of the participants.

Even this protocol work at our church. I have wholeheartedly accepted to be doing this work, so I enjoy. I do that work without considering what other people say. As you know, there are other people in a group who utter some discouraging words, but I started doing that work on my own and I enjoy doing it. Mw 24 Urban F 26

I: You have mentioned many things. So out of all the things you have mentioned, what things do you enjoy doing? P: In my life, I really enjoy worshipping God through singing in a choral group and spending time there. Sometimes they tell me to lead a song, pray, preach and teach. It thrills my soul because my breath of life belongs to God. Mw 21 Urban F 58

If someone who has money is not in good health, there is nothing that he can achieve, because there are certain things that require personal control. So, for someone to control all those things, he need to first look at his health. Thereafter, it’s when he can look at those other aspects. I mostly prioritize on my health by bathing, properly washing my clothes, drinking safe water and choosing a good diet, not just eating every food. In so doing, it means I am having a quality life. I shouldn’t also have stress because stress is an indication of not having quality life. When someone is having a quality life, his mind is supposed to be free. Having a free mind is an indication of having quality life. Interaction also helps someone to have quality life. Mw 24 Urban F 26

I like choir, going to the church so much, singing in the church’. Tz 31 RURAL F 57

The other community activities that were a source of enjoyment for the participants were wedding ceremonies and parties. In a way, they were also a part of supporting each other.

I: Is there anything else that you enjoy doing? P: The other thing I enjoy doing is the issue of wedding ceremonies, as you said. I enjoy attending wedding ceremonies for my friend’s children. We enjoy because the child of our friend is getting married. Mw 25 Urban F 63

I: Activities like parties, problems in the community? P: We cooperate on parties, if its a party we go and work together and contribute till we finish on the evening its when i get back I: Other activities that you attend for example death? P: If someone dies, we have a habit of cooperating with others in case of death when one has dies you have to go and cooperate with them to have strength to see that they have people. Tz 33 RURAL M 65

I: How does your friends and neighbours contribute to your quality of life? P: My friends and neighbours don’t contribute much, but little things. We sometimes agree to contribute money so that we can have a party. So such kind of things. Mw 34 Urban M 30

One participant mentioned taking part in charity works as a source of enjoyment. She describes the feeling she gets from helping other people as having achieved something as big as saving another’s life:

I: What other things do you enjoy doing apart from religious issues? P: I sometimes spare time to cheer the sick in hospitals or at home, do charity works and encourage our friends who are sick. Some of them have hypertension, but they don’t manage to go to the hospital. So we teach them. I: What really gives you pleasure when cheering the sick? How do you feel? P: I feel that I have done a good job because I have encouraged them and saved someone’s life. Mw 21 Urban F 58

## Taking part enables ideas to be shared

The other positive aspect that the participants talked about was that being able to participate in community activities enables for the exchange of ideas to take place. From some of the participants’ accounts, they talked about the exchange of ideas that happens during people’s interaction in groups. These ideas range from business ideas to farming practices:

What things do you enjoy doing in your life? You have said that when you wake up in the morning, you sweep, prepare food and do other things. You also attend wedding ceremonies and visit your relatives. You have explained different things, so what things do you enjoy doing? It can be things that you have mentioned or those that you haven’t mentioned. What things do you enjoy doing? P: I can’t solely depend on myself, so I enjoy staying in groups with my friends, so that we can know each other. During those interactions, we sometimes tell each other to do a certain income generating activity. Mw 23 Urban F 40

I: What else do you enjoy? P: I like to learn something that I don't know for example I can get to someone's place and look at may be a farm is good I wish to ask on which method was done to be like that or I look at livestock there are good and breeding so much I wish to ask what have you done to be like this, wither looking at the environment and like it I will ask what has been done, things that are surrounding us but I like them for good not forcing I wish to be like them, I wish I had a big ability to do whatever I want but some you fail to achieve because of your income and the situation you are in but we move slowly to imitate the good examples on the environment surrounding you. Tz 29 RURAL F 45

In the quote below, the participant is talking about groups called Chineba as well as village banks. On top of the emotional support from these groups, there is also an economic element:

I: Do you assist one another whenever one of you is having a minor or major problem? P: Yes, we assist each other. We have formed some small groups that we call Chineba. I have joined two Chineba groups. When I went to construct the sepulcher, one of the Chineba group followed me there. My fellow members of village bank also visited me. So I was pleased with this and this is the way to go. Mw 19 Urban F 45

In the final two quotes, we see the church acting as both a moral compass as well as a community that enables for the exchange of ideas:

How does your spirituality and religion contribute to your quality of life? P: In my quality of life. Sometimes what happens is that, in reference to decision making. When I go to church, fortunately I find that someone who is preaching that day touch on that part. When he says that as Christians, we are supposed to do the following things, it guides me. Church is a place where I build up relationships because I meet a lot of people from various sectors, whom I never knew. We learn about our relationship with God as well as how to relate with our family members and relatives. That is the important of attending church service. To uplift my spiritual life so that I can live rightly. Sometimes we think of doing a certain thing, like shouting at our friend, but we hear about forgiveness at church. So it’s part of our inspiration. Mw 27 Urban M 69

I: How is your spiritual life? P: My spiritual life. In the past, I was stranded. I had nothing to do and I stopped going to church. I: Was it after you left your father’s house? P: After I already left and was staying here. So my fellow choir members visited me. They encouraged me to be going to sing choir, on the set days. So I started going there. As I was going to the church there, I was really enjoying when singing choir with my friends and even on Sundays. In the course of that enjoyment. My friends told me that it’s better to start shares. So I started shares and when we raised some money at shares, I borrowed it and start doing business. I: So the church gave you an opportunity to know about shares and borrow money? P: Yes. Mw 12 Rural F 29

## Taking part brings social standing

The other positive aspect was how taking part in certain community services brings social standing. From the quotes below, it is clear that it is mainly the church that provides social standing from positions of leadership:

I: You have not told me about the church P: I am a leader in a church, I am Sunday school teacher so all that is mine, there is time to get prepared to talk to children like I have said resting it can be used to prepare what to teach, i get prepared in the afternoon. Tz 27 RURAL F 50

I don’t hold a position for the main church, however at our youth department I have one. I am the chairperson of the food committee. Mw 3 Rural M 25

Yes. I have served different position in Roman Catholic Church. I was the chairperson for our choir for a long time. Mw 4 Rural M 67

I: You sing in a choral group at your church. Do you hold any church position apart from singing in a choral group? P: I am the choir mistress. I: Choir mistress, you lead the choir? P: Yes, I am the leader. I: You minister in that position? P: Yes. I: Are you satisfied with that position? P: Yes, I am satisfied with it. I: What actually makes you to be satisfied with that position? P: It’s God’s work. Mw 18 Urban F 30

The participant below provides another opportunity for social standing through the position of leadership in the community developmental activities:

From there, I was elected chairman for MCPC. Thereafter, since I was ageing, they couldn’t just leave me without any leadership position. So I was finally elected as chairman for developmental activities. Mw 4 Rural M 67

## Other positive things about taking part

The accounts on the other positive things about taking part in community activities were on the community as well as individual benefits from developmental activities:

It affects us because if there are good roads in our village, cars and bicycles move properly. Mw 6 Rural F 27

I: Do you enjoy taking part in developmental activities in your village? P: It’s good to undertake developmental activities because it helps the village. If there is a pothole on the road, we fill it, so that we can walk properly. If there were some stones on the road, we also cover them with soil. Mw 15 Rural M 26

I: Do you enjoy taking part? What do you do as a way of participating? P: I pay money for developmental activities so that children should learn properly at school. I: Do you pay as a household or maybe individually? P: When the people are collecting the money. I: How much do you contribute? P: K1000. I: Are you happy with the issue of paying money for developmental activities? P: Yes, because the children are learning. Mw 14 Rural F

## NEGATIVE aspects of community

A small number of the participants from Malawi talked about the negative aspects of community. The participant below complained about the corruption among community leaders when it came to funds for development activities:

I: Do you take part in social activities happening in your village, like developmental activities? P: I take part in developmental activities, but, as of now, the thing that made me not to take part is that chiefs embezzle the money. I: Can you explain? Which money does chiefs embezzle? P: Chiefs have this problem. They send someone to proclaim that they will be collecting money for developmental activities. After, we have contributed the money, they don’t use it for the intended purpose, they put it in their pocket. I: How much do you usually contribute? Do you pay individually or as a household? P: As a household. This time they raised it to K5000, but we haven’t yet started contributing because we claim that the money is too much. I: When you contribute money in the past, what were the chiefs mainly using it for? P: If funds have been provided to construct certain structures, that money was paid to builders and male labours who were do the mixings and assisting in construction work. I: Constructing structures at the hospital or maybe at school? P: At school. I: At Chisumbu primary school? P: Chitende primary school. I: So you are not satisfied with how the money is used? P: Yes. I: Have you ever taken any step to inform the authorities as villagers? P: To be honest, I haven’t done that. I: Is there someone who take part in developmental activities, who think like you that chiefs don’t properly use the money or maybe you are the only one? P: It can be many of us, but I don’t know what others think. Had it been that we discussed this during a public gathering that chiefs doesn’t do well, so we shouldn’t contribute money, it’s when I would have told you that we are many. As of now, I don’t know about other people. I: Has your friends paid that K5000? P: I don’t know, if some have paid or not. If some of them paid, they would have come to collect the money. Mw 16 Rural M 41

Other participants talked about unpleasant interactions with their peers even those brought on because of social standing:

I: What factors contribute to poor quality of your life? Quite the opposite. P: For me to have poor quality of life is when something disappointing has happened and I am not pleased with it. In so doing, I become disappointed and the person who has disappointed me regard me as a bad person. Mw 25 Urban F 63

It can be the state of affairs in our country or about your family or in your village. What are the opportunities and freedoms that you value? P: I like that question because I normally think about it. You know, in life there are a lot of things happening, bad or good. You don’t expect in life to live a good life all the time. There can be a certain friend who can blame you that you are not doing well. Sorry to say this, as an example. I was the treasure for this village. They were bringing money to me, so I was properly recording, writing reports and deposit the money at the bank. But somebody within the vicinity could look at the whole thing and say that he suspect me of eating the money. So it’s a delicate issue to point out a finger to everyone within the locality or say that your friend doesn’t go to church or other things. While somebody is also blaming you. Mw 27 Urban M 69

Some of the participants talked about the bad behaviors from members of the community:

I mean doing unnecessary things in our lives and there are many things. Sometimes, we as men like having sexual relationships with our friend’s wives and this is not good enough. Someone can end up getting killed there. Mw 4 Rural M 67

In addition, its disobedience to God’s commandment. There is a commandment that “Thou shall not commit adultery” so it’s related to this. It’s one thing that a person need to be cautious in his life. Furthermore, stealing someone’s properly is also a bad behavior. Mw 4 Rural M 67

# Faith and Spirituality

Expressions of faith as important for a good life was clearly expressed by participants from both Tanzania and Malawi. Attending church and praying throughout the day it has made them to feel good and their lives has been meaningful.

It gives me peace when I thank God, go to worship and come back and I am at peace when I hear the word of God I really rejoice even when I am sick I feel light/better. Tz 8 URBAN M 64

What I enjoy so much in life ever since I was a girl is worship because it keeps me close to know God and all I do becomes successful, worship life I enjoy more than anything because it rectifies me when I am wrong, I have lacked anything at all because of the life I have lives even when I face challenges, cow dying but I keep telling God if you have brought this you know the way, when you struggle there are those who hate that, I enjoy what God does to children that is why I enjoy’’ Tz 27 RURAL F 50

Some stated the need for prayer before going to work or travelling that given faith that God blesses an activity that is prayed for, making a way whenever there is hardship with a belief that after prayer it will be easy for one to earn a living.

P: Attending church prayers is very important. I have stopped fellowshipping with my fellow congregants, but I pray on my own. When I am about to leave my house, I pray that God should make me to travel well, when going to my piece work. After I have prayed, it’s when I go to work. Mw 16 Rural M 41.

P: Its important to engage in religious activities in praying to God whenever there i hardship God should make it easy and that is when we earn a living that God set for us, that is what helps you pray to God. Tz 7 URBAN F 62.

Without forgetting the fact that God is everything and that He is the one who gives a way even during the time of hardship, when one prays there is a belief that difficulties will be made possible to go through including working hard after prayer things becomes possible.

P: They are godliness issues. I know that it’s God who makes everything possible, so it’s part of my spiritual life. Even if, I can face any kind of problems, but when I remember that God loves me, it gives me inner peace. Mw 18 Urban F 30.

P: Another thing is praying to God as you know God is everything, you know when you pray to God and work hard your life will be okay. Tz 30 RURAL M 26

Faith was valued for offering a moral compass amongst young and old alike

I: What things do you enjoy doing in your life? P: Praying, whatsapp and Facebook. I: What do you really enjoy about praying? P: We are supposed to pray in our lives so that we should be encouraged when weak. When we ask God, He answers us. I get encouraged and I can’t do sinful things, when I pray. As I said, I only stay with my child, so I face a lot of temptations. So when I pray, I can’t engage in those things. Mw 29 Urban F 33

And was synonymous with a good life

I: What else is needed for someone to have a good life? P: For someone to have a good life, she should love God. When a person loves God, things work well for her. When we cling to God, He assist us. Mw 32 Urban F 68

I: Another thing? P: Another thing is praying to God as you know God is everything, you know when you pray to God and work hard your life will be okay. Tz 30 RURAL M 26

# Health

Being healthy was a frequent response when participants were talking about both good and poor quality of life. As well as valuing health because it is needed for financial security, it was valued in its own right, as a component of good quality of life and poor health was seen as contributing to poor quality of like. Access to health care was also seen as important for a good quality of life and people took some actions themselves to stay healthy.

## Good health was highly valued

Good health was valued by people in both Malawi and Tanzania, by both women and men, in urban and rural areas, with and without chronic illness and of all ages. It was a frequent response when talking about both good and poor quality of life. For some, just being alive was an aspect of life they valued. As this 65-year-old man said, he was just grateful for every day:

I: What do you enjoy in your life? P: I enjoy mostly sitting and waking up in the morning and thank God that thank you for another good day that I did not expect and all the time you are asked to thank God all the time. Tz 33 RURAL M 65

Wellbeing and feeling strong and healthy was celebrated, as this woman reported when asked what she enjoyed:

P: For sure i enjoy wellbeing I: Why do you enjoy wellbeing, what happens? P: I enjoy, even when i wake up when i go to the market, i thank God to whatever i sell and get happy and thank God for waking me up strong and healthy I: Is there anything else that you enjoy in life? Tz 15 URBAN F 40

What i enjoy and thank God is that I am healthy, I have no pain anywhere, I can do the activity I want with no problem that is what I enjoy in life. Tz 7 URBAN F 62

Good health was seen as a basic necessity for a good life for example:

I: what do you value in your life? P: I value first it's my health it has to be good, second I value work that I do because that is what gives me life, three I value my life so very much over anything else it's a priority. Tz 29 RURAL F 45

so a good life is supposed to be healthy and enjoyable. The key thing is about having good health (participant laughing). Mw 19 Urban F 45

I: What do you consider as basic necessities for good life? P: The first is good health, money because with no money there is no good life I: How an health contribute to quality of life? P: With good health nothing will be hard you will struggle on everything, so health first. Tz 14 URBAN F 63

Equally, poor health was seen as contributing to poor quality of life:

The other thing that contributes to poor quality of my life is when someone feel sick. Mw 5 Rural F 40

Good health was also seen as the absence of disease and illness. Listing what made a good quality of life this man said:

Thirdly, living a life free from sicknesses. If he doesn’t have diseases and he is energetic, it brings high quality life. Mw 4 Rural M 67

I am satisfied because I don’t feel any pain in my body. My body is just fine. Mw 7 Rural F 60

I: What do you enjoy in life now? P: I thank God that in health, nothing disturbs me so much like diseases so the issue of health if comes a person gets confused, you have to check on the reality of life. Tz 17 URBAN M 70

Some distinguished between good bodily health and the absence of stress, for example, asked about her health one woman said she was stressed but said her physical health was good:

My health is good because even i did check up its good, but there is stress because of how life is going, but physically its good. Tz 1 URBAN F 45

For some, eating well and a balanced diet was a pre-requisite for good health, seen as synonymous with a good life and health:

I: What are the factors that bring quality to your life? P: The factor that bring quality to my life is eating a balanced diet, like nowadays. We eat vegetables, fish and meat sometimes. This makes my body to look healthy. Mw 33 Urban M 68

I: You have talked about eating a balanced diet. So how does this bring quality to your life? P: When I eat a balanced diet, I rarely feel sick. Mw 23 Urban F 40

## The impact of poor health

The impact of poor health on income or earnings has been discussed in the previous section, in financial security. In Malawi, though not in Tanzania, participants discussed other negative impacts of poor health. So just as good health was valued, poor health was seen as very detrimental to quality of life.

Poor health was said to bring stress, anxiety, or ‘lack peace’ for example, this woman with diabetes was worried when her blood sugar was not in the normal range:

When my sugar level is high or very low, it makes me to lack peace because it’s not supposed to be like that. My sugar level is supposed to be found normal when tested, it’s when I can feel comfortable in my life. If I sometimes feel better, it makes me to have a good life and I feel relieved in my body. On the other hand, when I am sick, it becomes a huge burden and I even ask myself questions that ‘why am I suffering like this? Why is this clinging on me like this?’ Some of my friends also suffer from diabetes, but they live a good, strong and healthy lives. ///// Most of the times, I lack peace, joy and happiness in my life. Mw 28 Urban F 51

When asked what contributes to poor quality of life, another answered:

The contributing factors for poor quality of life are sickness, poverty and lack of money. I: Can you shed light on that? P: When a person is sick, she usually gets worried because she feels pain the whole body. So, she usually feels anxious because of that sickness. Mw 21 Urban F 58

And this older man explains that illness means he is ‘troubled in his heart’:

The thing that I am not happy with in my life is the problems I have in my body. I am not happy with this because I live a miserable life. My sickness is what makes me to be troubled in my heart. I contemplate that how can I feel better or why are my friends living healthily. Mw 1 Rural M 80

One of the main reasons poor health was said to be difficult was that it prevented people doing what they wanted to do. For example, growing vegetables as this older women explained.

At the moment, I am not in good health because I suffer from diabetes. Both me and my husband suffer from diabetes. We don’t cultivate crops the way we used to do, in the past. As of now, my children just give us money for paying labours who work in our garden. We don’t cultivate crops the way we used to. We used to cultivate a lot. Mw 32 Urban F 68

Participants talked of ‘failing’, of not managing to do what they wanted to do:

The things that are important have been changing because of the disease. I don’t manage to do certain things the way I want, I fail. Sometimes when I work very hard, it’s when my sugar level starts going higher. So it prevents me from doing what I wanted to do. It becomes difficult for me to properly do that. Mw 28 Urban F 51

And not being able to do what one used to be able to do

Yes. Have they changed as you are aging? P: They have indeed been changing as I am aging because I find many things on my own. I am aging, lacking strength and approaching old age. There is a difference in the things that I used to do when I was still strong, as compared to this present time. This is why there is also a difference in the things that I used to find in the past as compared to this present time. Mw 25 Urban F 63

Two mentioned having to take medication as contributing to poor quality of life. For example, this participant talked about his reluctance to take medication for his blood pressure, thinking about managing it through herbs or physical activity, but in the end having to accept medication:

Maybe, hypertension is the only part of the poor quality of my life (participant laughing) because I have to keep on taking medicine and the like. People have been talking about herbs. People say that sometimes when you do physical exercises and all those kinds of thing, this will go away. I have tried but it looks like it’s not really going away. I have also tried using herbs. You know when you fight something, it really pains more. You just really have to accept it that fine, this is the situation which I am, I have hypertension. Let me try to just live with it, accept it, manage it. Wherever it will feel like this is the end, let it be there. But at least by the end of the day, trying to fight something which you cannot change, I really feel it’s one of the greatest misery that you can live in. Mw 31 Urban M 49

One participant felt that ill health comes to dominate all aspects of life, including how you think about someone:

It’s not good for someone to feel sick because the mood changes. When someone is just okay, we can be chatting, laughing and doing other things, but when she is sick, I get confused and my way of thinking changes. I feel sorry for her and think about the disease that time. Mw 30 Urban F 28

## What keeps people healthy?

Three people, again Malawians, talked of keeping physically active as important for health. This older man mentioned walking around his house to keep active, and also going to his children’s land to help cultivate vegetables to keep active:

In reality, God made us so that our bodies shouldn’t be dormant. //// Of course, my children have farming land in [name of place], so I go there once in a week or once in two weeks and so on. I go there and move round to monitor what is happening around there. I feel that my fitness can be in place when I at least do something, instead of just sitting on that chair, when I wake up in the morning, up to 8 hours watching TV, I don’t believe in that. I believe that there is some fitness in me because I don’t like staying idle. ////. But for me stay fit, I just move around. So, I do those things. Mw 27 Urban M 69

And one woman explained the importance of moving early in the interview when she explained what she did in a day:

When I wake up in the morning, I pray. After that, I go outside to sweep. I don’t depend on my children. My children are supposed to do other things and I also do some work so that my body should be strong. If I just stay idle, it means I am not doing physical exercise. If I do household chores like washing clothes, cleaning plates, sweeping in my bedroom and sort out things, it’s when I feel better. Mw 21 Urban F 58

## Access to health services and medicines

Health services and medicines was seen by many as an important source of good health in both countries, by men and women in all settings and with and without chronic illness. One older rural participant said simply that he was pleased to have health and other services available:

I: Is there anything that you enjoy? P: Its health services being close to people together with government offices that are surrounding us, a problem can arise and you get to them to explain. Tz 33 RURAL M 65

And also explained that he thought health services were a basic need in life:

I: What are basic necessities for good life? P: To have hospitals, school, to have food at home, to have water at home, farming, market close to the people I: How about a house? P: Having a house I: How about clothing? P: It’s a basic necessity. Tz 33 RURAL M 65

A more common theme, amongst those with chronic illness and those without in men and women and in both settings was that the cost of health services was a worry. This participant wanted health insurance for all. When asked what makes a good quality of life:

It’s having a treatment stand especially at times you might not have money, so health insurance is important, without health you cannot work. Tz 28 RURAL M 67

In Tanzania, the discussion focussed on the cost of health care itself, for example, if you do not have insurance, you cannot afford care:

The situation I am seeing like me, the big thing is hospital services you find that you have no insurance, you have no money you think till you get tired on your head. Tz 8 URBAN M 64

Other participants explained that health care should be free for children and older people through government-paid health insurance but in practice that was not delivered:

I would like to see changes in health, especially in hospitals. If health services were improved because, yes, we are told that for children and old people there are drugs, but these things are not done, we wish that they are done. If a child under five years is taken to the hospital with a certain need it should be done. Tz 6 URBAN F 33

She went on to explain that even if people bought health insurance, they find it does not cover the full costs of care:

There is this issue of health insurance, this insurance that we pay for we would like them to work really well. For example, I have paid health insurance for a child of a certain amount, I will not mention people's companies, an amount of 51,000/=. Then when I go for treatment, it should be covering those diseases, or these processes should be clear so that the services should be free. //// and in health in the hospital old people’s and children’s drugs should be there and they should be taken care of. Tz 6 URBAN F 33

Another Tanzanian participant said the same thing and went on to mention that the costs of drugs is prohibitive:

Aspects that contribute to poor quality of life? You know you have to fight three things, disease, ignorance and other things. But for now, the reason from the healthy policy it says that 0 to 5 years the service is free, but such services are political it’s not real. And if there are diseases like diabetic victims when we go to clinics there are no drugs you have to contribute and other people cannot afford to contribute, many don’t afford to contribute so the government is still a challenge because many are complaining, people are dying because of lack of service that is what contributes to poor quality of life. Tz 5 URBAN M 49

The actual availability of drugs as well as their cost was also mentioned as a problem in both countries, but with even more examples from Malawi. For example, one person said that poor access to drugs from the health service contributes to poor quality of life:

So those things contribute to poor quality of people lives and I would love to see those things change. There should be availability of medication in hospitals. Mw 20 Urban M 48

And this older man with a chronic illness explained that drug availability was a problem:

I can say that months have passed. When I go to the hospital, they say that they have run out of this medication. Mw 8 Rural M 73

And a younger man reported being to actually afford medication was a problem, which could leave someone feeling anxious:

I: What do you mean by good health? P: Rarely becoming sick. If I suffer from a disease, there should be a possibility of accessing better treatment at the hospital. I: What do you mean by better treatment? P: Okay, better treatment for me. When I go to the hospital, I shouldn’t feel anxious that I might find that there is no treatment. For instance, if I feel like having malaria and have gone to access medical support. They shouldn’t just say that I have been diagnosed with a certain disease, but there is no treatment. That I should buy [the medication] at a pharmacy. They inform us those things at a time when we don’t have money for buying medication. As a result, people lose their lives because they didn’t receive treatment for their diseases at an appropriate time. Mw 22 Urban M 30

Two people also mentioned the waiting time at hospitals as a problem. This younger, healthy, participant felt sorry for the older people who were left waiting a long time. Asked what would improve quality of life he said:

Hospitals, like elders, there should be nurses. [When] I went to pick up drugs there was one nurse. /// I came at eight and left at ten, looking behind there were many elders in line. I felt bad that I am getting [served] and leaving them behind. There should have been another nurse in the other room and if a patient gets in, he will spend like 6 or 7 minutes [not 2 hours]. Tz 24 RURAL M 18

Another female participant mentioned the same thing when asked what changes she would like mentioned the opportunity costs of spending time waiting for care:

I would like changes on, everything to be done on time. For example, you have gone to the hospital you get service on time so that you get back to work and not going to the hospital in the morning and getting back home at two there you cannot work. Tz 25 RURAL F 55

# Autonomy

Under autonomy, the participants talked about the ability to make decisions for their lives as well as that of their family. From their responses, they talked about both having the ability to make decisions as well as not having ability to make decisions and how the two determine one’s quality of life.

## Having the ability to make decisions

The responses on having the ability to make decisions were more common among the Malawian participants as compared to the Tanzanian participants. There was no variation noted in the responses according to gender, age and setting. Some of the participants talked about being able to make decisions on their own, independent of other people’s influence. Below, are a few examples of such participants:

I: Whenever you want to make a huge decision, do you personally make the decision or maybe you consult your friends? How does it happen? P: I personally make the decision. I don’t consult anybody. Mw 25 Urban F 63

I: What else is contributing to quality of life? P: May be what contributes to quality of life is making my own decisions not doing something for someone's benefits. Tz 12 URBAN M 30

I: How about ways to make decision? P: I see like now I have a bigger capacity to make decision compared to the past, because i had to ask for permission, get advise at times you are told not to go but now I can ask why should i not go, you tell him I am going because of one, two three I: Why do you think of these changes? P: may be after seeing I am old and i am aware that I can make decision, for example there is no school fees I am going to the group to lend some money for school, I know that is within my ability to decide he can ask you on how it will be paid, a way will come later. Tz 25 RURAL F 55

You started mentioning when you said that some people when they look at you they say you are well-to-do. What are those things that you feel contribute more to your quality of life? P: The best thing that I am looking at in myself is “live a life of yourself”. Like in my case, as said, (I was waiting for the motorbike to pass because you are recording). I am not really in a situation where I am competing with anyone else. In tumbuka language they say “Mbuzi yakugolontha yikujimanya yekha.” What I would really want to interpret is that, if you are herding goats and one of the goat has got maybe a limp or maybe it’s not feeling well, it will not really try to compete with the others. It will not be eating and eating until 04:00 and decide to go back home. It will know that I have eaten and because I am having a problem of walking, that is “kugolontha” let me start off a bit earlier than my friends so that I reach home in good time, as well. So what I am trying to put across is, I have learnt or train myself to live a life of who I am or who I want to be. So at the end of the day, irrespective of what other people do or what my friends do, I still continue doing what I feel is the right thing for me to do. That has been one of the major thing which I feel has really brought me peace of mind. Mw 31 Urban M 49

From the participant above, we can get a clear sense of how much he values autonomy. He is able to articulate how much his ability to make decisions that are right for him has contributed to his quality of life.

A majority of the participants mentioned consulting family and friends in their process of decision making. In their accounts, they highly valued the other people’s input:

P: I personally make the decision sometimes, but if I am finding it difficult to make the decision, I ask my friend on how I need to do it. I: If you are pleased with your friend’s opinion, you follow it. If your friend’s opinion is contrary to what you wanted to do, do you still do what you wanted to do or maybe you follow your friend’s opinion? P: I follow what my friend has told me. Mw 23 Urban F 40

I: What is your approach to decision making? When you want to make a certain decision, do you personally make the decision or how do you go about it? P: When I want to make a decision regarding a certain thing, I first weigh it to see how I will benefit from that thing or problems that will come about. When I contemplate on that thing and fail to make a decision, it’s when I consult someone. That person can’t be suitable for that thing, but I try my best to consult someone who can give me wise and sensible advice that can help me. If I fail to make a personal decision, I first consult my guardians. I tell them that I have these plans and this is what I am thinking, so how can you assist me. They don’t make a decision for me, but they enlighten me that with how things are here, if I can do it in this way, these are the problems that can come about and these are the benefits, so how do I look at it. From there, it’s when I contemplate on how to do it, basing on what they have said. Sometimes, I also ask my friend that there is a certain thing, so how do you look at it. I don’t ask him in order for him to tell me what to do, I just ask him so that I can hear his point of view. If I compare his point of view with what I am thinking, it’s when I conclude that this can be the right thing. So most of the times, I make personal decisions. Someone doesn’t make decisions for me, but I make decision after I have made a research on what I can benefit. If I discover that this is what I will benefit, it’s when I make a decision that I need to do it this way. Mw 24 Urban F 26

I first make a personal decision. Thereafter, it’s when I ask other people that I have come up with this, so how can I do it. I first make a personal decision and after I have contemplated about it, it’s when I consult other people for further assistance. Mw 6 Rural F 27

I: In terms of making decisions in your life. If you want to do a certain thing in your life, do you consult other people or maybe you personally make the decision? P: I consult other people. I: Which people do you consult? P: My mother. I: Do you consult your husband? P: I also consult him. I: You also consult your husband. Does consulting people regarding a certain thing that you want to do pleases you or not? P: It’s important. I: Important in what way? P: Because people give their opinion on that. If they feel it’s not a necessary thing to do, they also inform me about the advantages and disadvantages. Mw 18 Urban F 30

Some of the participants highly valued the family unit in their decision making process:

I: Whenever you want to make a huge decision, do you personally make the decision or maybe you consult other people, like discussing with your wife before making the decision? P: Since I got married, I usually involve my family when making decisions. I inform my wife that I would like to do a certain thing, so I ask her questions, so that she can also give her opinion. I: Okay, you don’t personally make the decision. P: No, when I have come up with a certain issue, I am supposed to ask her if it’s helpful for us to do that. So that she can also give her opinion. I: You only consult your wife? P: Yes, I don’t have time to ask other people, apart from my wife. Mw 20 Urban M 48

I: When you want to make a huge decision that will completely change your life, do you personally make the decision or maybe you consult other people? P: I consult my husband at home. When we sat down, I told him that we are approaching rainy season, so what should we do. So both of us gave our views, that due to shortage of money, we won’t manage to grow maize, so what should we do. We finally agreed that we should just grow groundnuts. So this is what we sometimes discuss. Mw 21 Urban F 58

What is your approach to decision making? Do you personally make the decision? How do you come to a decision? Do you consult your wife or friends or how does it happen? P: That is another good question but a little bit tricky. Anyway, decision making, if all of us are available starts in the family and I can’t ignore my partner or wife. I can’t just decide that we should move out of the house so that someone should be renting it. I can’t be assisted and God can’t guide me. Decision making starts in the house. Thereafter, it’s when we consult our children, if they reside close or at a distant. We inform them that we want to do a certain thing, so the children can direct us. My wife can sometimes disagree or agree with the decision that I want to make. That’s the best way of life. So it starts in the house, especially with my partner, if we are both alive. I can’t ask our chief about a decision that I want to make in my house. If I can ask him that I want to do a certain thing in my house, he can just look at me and say that this person has really grown old. Mw 27 Urban M 69

I: How about having freedom on decision making? P: I make decision with my children. I have decided that I will take care of my children alone because of their mother, if I decide to do something different even my God will beat me I: Why? R My wife was respecting me so much, deciding to have another wife will be a different decision, we an agreement with our wife that even if I die or she die today I will not get married, I also told her if I die she should not get married again, so I have to adhere to our agreement because she is the one who is no longer alive. I will continue taking care to my children until when I die. I will never marry another wife again. Tz 19 RURAL M 35

I: Whenever you want to make a huge decision, how do you go about it? Do you personally make the decision or how does it happen? P: No, we don’t do many things on our own, we tell our children. We discuss with them, whether issues about fertilizer, farming activities or things that are happening here at home. We inform the children, so the children discuss this separately and agree on what to do. Thereafter, it’s when they tell us what they have agreed. So there is a very good relationship. Mw 32 Urban F 68

For some participants, making decisions on their own was not a choice but a consequence of certain circumstances. For example, this 33 year old female has had to make certain decisions on her own as a result of a divorce:

I: What choices do you make in your life? What freedoms or opportunities of doing things do you value? What opportunities do you have of doing things or certain kind of freedoms that you value? P: God has given me an opportunity of life. In terms of freedoms, I don’t know which freedoms I have, is being single freedom. I: Is it not freedom? P: No, it’s not freedom. It’s just that things didn’t work out, but I never wanted to remain single. It just happened that things got out of hand and I have ended up becoming single. I thank God that I didn’t go to the village. When many women divorce here, they go to stay with their parents, but I refused that kind of life. I couldn’t say that I was going to the village because this would have impacted on my child’s education. My child was learning at a certain school when my marriage ended, so she continued to learn at the same school until she wrote her PLSC exams there. Had it been that I went to the village, my child would have started learning at a government school. So I refused that. I learnt school in a difficult way up to form 4, but I want my child to be educated. I have this opportunity and I thank God for giving me wisdom for doing business, so that my child should continue with her education. Mw 29 Urban F 33

## NOT having ability to make decisions

Only one participant from Tanzania described not having the ability to make decisions as a hindrance to achieving goals in life.

I: Issues related to decision making, do you think it's something that can contribute to poor quality of life? P: Yes I: How? P: It's when you want to do something but you have no ability to do that, so that brings stress on how to solve that already you don't reach the goal. Tz 32 RURAL F 45
