# Supplementary material for: A more equitable approach to economic evaluation: Directly developing conceptual capability wellbeing attributes for Tanzania and Malawi
Source: Soc Sci Med. Author manuscript; Available in PMC 2024 Nov 6. (PMC7616778; doi:10.1016/j.socscimed.2024.117135)
Supplement: Appendix A. Supplementary data [file EMS199746-supplement-Appendix_A__Supplementary_data.zip › 1-s2.0-S0277953624005884-mmc2.docx]

**Supplementary File 2. Capability Wellbeing in Tanzania and Malawi Coding Frame for final analysis of attributes**

We are trying to get mutually exclusive coding – there is potential for overlap between short term financial security and basic needs which we can try to avoid – see notes below

| Attribute – being able to have… | Explanation |
| --- | --- |
| **Having Financial Security**  DO NOT CODE THIS IN SOFTWARE PACKAGE – it is a descriptive heading, and the actual codes are below | The experience of *having* financial security  The challenges of living with uncertainty of financial resources available both currently and in the future  The ability to meet basic needs - the next theme/attribute - focuses on the immediate meeting of needs, this theme relates to financial security was concentrated more on the challenges of living with uncertainty about finances both currently, and in relation to the future and the avoidance of future shocks. |
| Having Short Term Financial Security | Reassurance that basic needs could be met  Knowing that there would be money available to meet their basic necessities in the short-term  Enabling them to provide for themselves. |
| Having Longer Term Financial Security | Enabling participants to balance between current spending and saving for the future, where savings could be used to invest in future wellbeing |
| Financial Security to avoid Implications of Future Shocks | Having access to resources to reduce the potential for stress associated with uncertain future shocks such as health expenditures.  Including the financial security to pay for insurance, and the financial security to pay directly for care. |
| **NOT having financial security**  DO NOT CODE THIS IN SOFTWARE PACKAGE – it is a descriptive heading and the actual codes are below | The experience of NOT having financial security |
| Implications of NOT having Short Term Financial Security | Being uncertain about their ability to meet needs now or in immediate future for themselves and/or their children which contributes to poor quality of life |
| Implications of NOT having Longer Term Financial Security | Not being able to invest in children’s education or in businesses |
| Implications of not being able to avoid Future Shocks | Not having financial security leading to anxiety about potential shocks that would reduce their ability to cope with life.  This anxiety contributing to poor quality of life. |
| **Sources of financial security**  DO NOT CODE THIS IN SOFTWARE PACKAGE – it is a descriptive heading and the actual codes are below | The sources of financial security |
| Business or farming prosperity | The success or lack of success of business or farming – having income from business or farming |
| Employment and wages | Having employment or wages |
| Working hard | Anything that is said about working hard bringing a good life (mainly because it brings financial stability) |
| Infrastructure | such as roads or electricity or water as a source of financial security (short term, for immediate needs or longer term) |
| Education | Education as a source of financial security, through being able to get better jobs |
| Children | The value of children was as a source of current (for older people) or future (for younger people) financial security |
| Health | The person’s own health as an important enabler to allow people to work and thus to earn or do well in business or farming. |
| Groups such as SACCOS or other micro credit organisations | Taking part in these groups as a source of income and thus financial security |
| Inflation as a source of financial INSECURITY | Anything said about inflation bringing financial insecurity |
| Climate change as a source of financial INSECURITY | Anything said about climate change and lack of water bringing financial insecurity |
| Other sources | Including, for example, wider family |
| **Being able to meet Basic Needs**  DO CODE THIS IN SOFTWARE PACKAGE – ALTHOUGH IT IS A DESCRIPTIVE HEADING OFTEN PEOPLE MAKE A LIST OF BASIC NEEDS AND WE CAN CODE THIS HERE | Lists of basic needs and anything said about ability to meet OR NOT meet immediate basic needs |
| Food as a basic need | The need for food (but note, sources of meeting basic need may be coded under sources of financial security above) |
| Electricity | Electricity as a source of meeting basic needs |
| Water | Water as a source of meeting basic needs (including hygiene, growing food and so on) |
| Shelter | Shelter as a means of meeting need for security |

| **Being able to participate in community activities**  DO NOT CODE THIS IN SOFTWARE PACKAGE – it is a descriptive heading and the actual codes are below | Taking part in community activities such as communal faith-based activities, weddings, funerals, sharing ideas with others and cooperating in activities with others in groups. |
| --- | --- |
| Taking part in activity brings enjoyment | Enjoyment in communal activities including playing or watching team sport |
| Taking part brings cooperation and support | Working in a cooperative group or getting support from taking part |
| Taking part brings social standing | Being respected and having a position as a result of roles in community activities including church or religious based activities |
| Taking part enables sharing of ideas | Sharing ideas through interactions with others in community |
| Other things from taking part in community activities | Anything else positive about taking part in community activities |
| NEGATIVE aspects of community, including bad neighbours, stigma of not having children, being HIV positive, |  |
| **Achievement and personal development**  DO NOT CODE THIS IN SOFTWARE PACKAGE – it is a descriptive heading and the actual codes are below | Ability to be able to achieve or develop for self and family |
| Being proud of what have been able to achieve for family or in life | Anything about pleasure, pride or contentment that have been able to achieve (e.g. in education, in farming in family) or provide for family |
| Being frustrated that have not been able to meet one’s goals | Anything about the frustration of not being able to achieve one’s goals |
| S**ources of achievement**  DO NOT CODE THIS IN SOFTWARE PACKAGE – it is a descriptive heading and the actual codes are below |  |
| Caring for children or family | Being proud/pleased that have been able to care for children well and be a good wife/husband  OR opposite – being disappointed cannot or would like to do more |
| Fulfilling plans for housing | Being proud/pleased that have been able to fulfil plans for housing  OR opposite – being disappointed cannot or would like to do more |
| Education | Being proud that one has achieved a good education or can provide a good education  OR opposite – being disappointed cannot or would like to do more |
| Working hard as a source of achievement | Feeling that one has worked hard as a source of achievement – that employment has gone well  OR opposite – being disappointed have not been able to work hard |
| **Health**  DO CODE THIS IN SOFTWARE PACKAGE – ALTHOUGH IT IS A DESCRIPTIVE HEADING we will code anything said about valuing health in its own right here | Ability to lead a healthy life, with health valued in its own right rather than as a resource for financial security  Including negative impact of poor health |
| **Sources of health as a value in its own right**  DO NOT CODE THIS IN SOFTWARE PACKAGE – it is a descriptive heading and the actual codes are below |  |
| Access to health care |  |
| Other source of health as a value in its own right |  |
| **Attachment, love and friendship**  DO NOT CODE THIS IN SOFTWARE PACKAGE – it is a descriptive heading and the actual codes are below | Ability to have and maintain attachments, including with close family (spouse/children), wider family, friends and neighbours |
| Interactions with / having **family** (including children, husband, wider family) bringing enjoyment and pleasure | Anything about the pleasure and enjoyment of having and maintaining love and maintain attachments with FAMILY - close family (spouse/children), wider family |
| Interactions with / having **friends and neighbours** bringing enjoyment and pleasure | Anything about the pleasure and enjoyment of having and maintaining love and maintain attachments with friends and neighbours |
| Interactions with / NOT having family (including children, husband, wider family) bringing stress and upset | Anything negative about interactions with or NOT having attachments with family |
| Interactions with / NOT having good neighbours of friends | Anything negative about interactions with or NOT having attachments with friends or neighbours |
| **Autonomy**  DO CODE THIS IN SOFTWARE PACKAGE – Although it is a descriptive heading we will code anything said about valuing being able to make decisions here | Ability to make decisions for their lives and that of their families, often in consultation with others  Include anything about feeling good that can make decisions with on own or in consultation with others |
| **Faith and spirituality**  DO CODE THIS IN SOFTWARE PACKAGE – Although it is a descriptive heading we will code anything said about valuing being able to make decisions here | Ability to express faith and pray though not the participation in communal religious activities such as going to church  Include anything said about pleasure/peace etc about religious practice BUT not participation in religious community-based activities like choir singing or church going. |
